# Associations between sunshine and influenza or influenza-like illness, a systematic review and meta-analysis

**DOI:** 10.1007/s00484-025-03121-0

**Published:** 2026-01-16

**Authors:** Savanna Ratky, Javier Chai Rui Cheng, Alexandra Schneider, Susanne Breitner-Busch, Annette Peters, Margarethe Woeckel, Regina Pickford

**Affiliations:** 1 Institute of Epidemiology, Helmholtz Center Munich, German Research Center for Environmental Health, Neuherberg, Germany; 2 Institute for Medical Information Processing, Biometry and Epidemiology (IBE), Faculty of Medicine, Ludwig-Maximilians Universität München, Munich, Germany; 3 Pettenkofer School of Public Health, Munich, Germany; 4 Department of Psychiatry and Psychotherapy, LMU Hospital Munich, Munich, Germany

**Keywords:** Influenza, Influenza-like illness, UV radiation, Sunshine, UV index, Solar radiation, Systematic review

## Abstract

Influenza and influenza-like illness (ILI) impose an immense burden to public health. Exposure to ultraviolet (UV) radiation might impact the spread of influenza. We examined associations between UV radiation and sunshine duration and influenza or ILI. We performed a systematic review and meta-analysis including studies with UV radiation, UV index or sunshine duration as exposure and influenza/ILI as outcome. A random-effects meta-analysis was conducted using the Knapp-Hartung method to calculate the pooled relative risks (RR) of the associations between UV radiation and sunshine duration and influenza. Several sensitivity analyses were performed. 45 studies which met the eligibility criteria were included in the systematic review, of which 16 were included for meta-analysis, covering more than 1,000,000 influenza cases. The pooled results showed every 1 W/m^2^ increase in solar radiation was significantly associated with a decrease in influenza incidence RR 0.996 95%-confidence interval [0.993 – 0.999]. In the meta-analysis of sunshine duration, pooled estimates showed no significant association between an increase in sunshine duration and influenza incidence (RR 1.003 [0.988 – 1.018]) per one hour increment. Results were robust for all sensitivity analyses. In this meta-analysis, an increase of UV radiation provided a protective effect, while sunshine duration showed no significant effect, perhaps due to lack of eligible studies on this relationship. Findings indicate that UV radiation could be a helpful parameter for predicting influenza incidence, specifically if other meteorological parameters such as temperature, humidity and wind speed and their interrelationship with UV radiation are considered.

**Supplementary Information:**

The online version contains supplementary material available at 10.1007/s00484-025-03121-0.

## Introduction

Seasonal epidemics of influenza are estimated to infect about 1 billion people per year, with 3–5 million of those as severe cases, and 290,000 to 650,000 respiratory deaths annually (Influenza (seasonal) 2023). The estimated economic burden of influenza is substantial, with $11.2 billion per year in the United States (Putri et al. 2018), $31.1–83.6 million per year in Thailand (Kiertiburanakul et al. 2020), and €78 million in Germany in direct costs per year (Scholz et al. 2019). Influenza has three strains that infect humans, A, B and C, with circulation of A and B resulting in seasonal epidemics (Influenza (seasonal) 2023). However, the seasonality of influenza C is unclear and relatively poorly understood. Influenza C is not known to cause epidemics or pandemics, and carries a lower disease burden than influenza A and B, making influenza C less epidemiologically relevant relative to influenza A and B. Nevertheless, influenza C virus remains an important respiratory pathogen amongst pediatric populations, as most primary infections occur in early childhood (Lee et al. 2025; Sederdahl & Williams 2020).

It is hypothesized that meteorological conditions such as temperature, relative humidity, wind speed, and UV radiation play a role in influenza and influenza-like illness (ILI) incidence and transmission. Chamber studies show that temperature and humidity can modulate the viability of viruses by affecting the properties of viral surface proteins and lipid membrane (Moriyama et al. 2020). Also, low ambient temperature and reduced humidity in the breathing air can directly affect immune defense against viruses thus making humans more vulnerable to viruses such as influenza (Moriyama et al. 2020). Epidemiological evidence indicates that extremely low levels of humidity are associated with the onset of epidemic influenza throughout the United States (Shaman et al. 2010). Non-optimal ambient temperatures have also been associated with chronic diseases (Fan et al. 2023; Liu et al. 2022; Moghadamnia et al. 2017; Phung et al. 2016; Zafeiratou et al. 2021) which might make humans more susceptible to influenza infections (Hovden et al. 2007; Maleki et al. 2023). Wind decreases air temperature and humidity and thus may impact susceptibility to influenza as described above. Wind also carries virus-containing air droplets and may therefore spread the virus (Dbouk and Drikakis 2020; Yang et al. 2021). Finally, ultraviolet (UV) radiation, has been shown to play a role in influenza and ILI incidence and transmission (Moriyama et al. 2020; Peci et al. 2019; Sagripanti and Lytle 2007). These meteorological factors are also interrelated. Measurements in Bejing, China show positive associations between ambient temperature and UV radiation, while relative humidity was negatively correlated with UV radiation (Liu and Zhang 2015).

For the relationship between sunshine/UV radiation exposure and influenza, two mechanisms have been proposed. First, UV radiation can cause skin cancer by damaging DNA in skin cells (Pfeifer 2020). Similarly, UV radiation is also capable of causing damage to RNA, such as influenza’s single-stranded RNA, and can lead to inactivation of viruses (Jensen 1964). Experimental studies have shown that exposure to UV light can render influenza viruses inactive by destroying their genetic material (Sagripanti and Lytle 2007). Viruses with a single-stranded RNA genome have been found to be more sensitive than viruses with double-stranded genomes to UV radiation exposure (Lytle and Sagripanti 2005).

Secondly, several studies have indicated that exposure to UV radiation modulates the immune system, particularly by affecting vitamin D regulation. UV radiation is categorized by its wavelength into UV-A, -B and -C of which UV-B plays the most important part for humans. Exposure to UV radiation increases production of Vitamin D in the human body which has been linked to a better immune response (Cannell et al. 2006) and bone calcification (*Vitamin D—Fact Sheet for Health Professionals*, 2023). Sun exposure is the most important source of Vitamin D because UV-B, with a wavelength between 315–280 nm, stimulates its production, by breaking bonds in 7-dehydrocholesterol in the skin, forming pre-D3 (Bikle 2000). The amount of pre-vitamin D formed is dependent on the amount of UV-B reaching the skins layers, and the skin releases Vitamin D into the body for up to 3 days after a single exposure (Holick et al. 1980).

A meta-analysis showed that supplementing Vitamin D was protective against acute respiratory tract infections, including influenza (Martineau et al. 2017), while another review found inconsistent evidence for these protective effects (Bryson et al. 2014).

Furthermore, climate change is expected to alter weather patterns, with extreme weather events expected to be more common (He et al. 2023; IPCC 2023). This could occur as heat waves, heavy rainfall, and other abnormal seasonal fluctuations and might also impact UV radiation, e.g. by increasing the number of sunny days. These effects of climate change could alter the spatiotemporal dynamics of influenza and ILI infections, raising concerns over the potential changes in disease burden of these respiratory infections under climate change (He et al. 2023). He et al. (2023) emphasised the need to fill the knowledge gap in achieving a comprehensive understanding of meteorological factors in epidemiological trends of respiratory infections including influenza and ILI.

Despite the proposed mechanisms linking UV radiation and influenza or ILI through in vivo and in vitro experiments, there is conflicting evidence (Park et al. 2020; Yan et al. 2024). Associations differ by climate region, as temperate regions have greater seasonal variation in meteorological factors than tropical regions (Charland et al. 2009). Epidemiological studies investigating the association between these factors and influenza and ILI often focus on specific geographical regions. Several reviews have explored the use of UV for influenza inactivation via irradiation in a laboratory setting (Glover et al. 2025; Hessling et al. 2022). Hessling et al. (2022) suggest that UV-A and UV-B require longer durations to achieve reductions in immunoglobulin levels, which in the environment we propose could be achieved through hours of sun/UV exposure. To our knowledge, this mechanism has not been assessed in a review at the environmental level. To build a comprehensive understanding of this complex relationship, a systematic review and meta-analysis is warranted to synthesise currently available evidence, and to identify areas that require further research.

## Methods

### PECOS

This systematic review aims to answer the following question: “In the general human population, what association does an incremental effect of a 1 unit increase in exposure to UV radiation, sunshine or UV index, have with the incidence, mortality, severity and clinical course of influenza/influenza-like illness?”.

Table 1 summarises the population, exposure, comparator, outcome, and study design (PECOS) statements for observational or human epidemiological studies. Corresponding elements to be excluded are also listed in the table, which informs the eligibility criteria of the systematic review. More details on the exposure and outcome can be found in the review protocol (supplementary materials S1).

**Table 1:** Population-exposure-comparators-outcomes-study design framework

*PECOS*	
*Population*	General human population of all ages, including those with certain health conditions and all socioeconomic groups
*Exposures*	Inclusion: - Sunshine/UV radiation/UV index - Short-term and long-term studies Exclusion: - Exposed in occupational/indoor setting
*Comparators*	Per 1 W/m^2^ UV radiation/1 h sunshine/1 unit UV index
*Outcomes*	Influenza and influenza-like illness (ILI) ICD 10 codes J09.X, J10, J11, J11.1, and ILI by symptoms
*Study Designs*	Inclusion: - Cohort, case–control, panel, case-crossover, time series, and any other epidemiological studies performed in humans Exclusion: - Human challenge trials, non-human in vivo studies, in vitro studies, reviews and other studies that do not present original data

### Search strategy

We searched PubMed, Scopus, Web of Science and MedRxiv using key terms “influenza” or “influenza-like” as outcomes with “solar radiation”, or “UV radiation”, or “UV index” or “ultra violet” as exposures. MeSH terms were used for key terms in PubMed to increase sensitivity to variations in terms or spelling. To include relevant publications with a primary focus on other meteorological terms, temperature, rain, humidity and wind were included in the search. Relevant meteorological terms for the search were discussed with the meteorologist (AS). We included long-term studies with exposure time-frames of at least 12 months, and short-term studies which consider shorter time periods, usually day-to-day variation. No date of publication restrictions were put in place. The search was conducted on May 10th 2023, with an update performed on October 21 st 2024. We also performed a grey literature search to retrieve additional articles from WHO, EPA, WorldCat, and Grey Matters from Canada’s Drug and Health Technology Authority (CADTH). Retrieved publications were imported to EndNote (version 20). The complete search strategies can be found in supplementary materials S2.

### Selection criteria and screening

After the removal of duplicate publications, three reviewers (SR, RP, and MW) performed title, abstract and full text screening in pairs to reduce bias. Studies were included if they met the criteria detailed in our Population, Exposure, Comparison, Outcome, Study Design (PECOS) framework (Table 1), had available full texts, and were in English or German. Discrepancies were resolved with discussion among the pair of reviewers screening the publication.

### Data extraction

A pilot study was conducted to test spreadsheets for data extraction for the systematic review and meta-analysis using three eligible publications. Once selected, information on studies was extracted by reviewers and input in the spreadsheet including: author, year, journal, study design, study population, study period, number of events, study location, exposure time frame (short term < 1 year, long term > 1 year), language, exposure measure, data source and assessment type (model or monitor), outcome measure, data source and assessment type (lab confirmed, ICD code, etc.), confounding variables adjusted for, statistical methods, confounder selection method, lag (short-term studies only), and effect estimates. Fields with missing information were marked as “no information”. In the case of insufficient information on statistical methods or when effect estimates of interest were missing, authors were contacted for information. An example of the spreadsheet used can be found in supplementary materials S3.

### Risk of bias assessment

For assessment of internal validity, we used the National Toxicology Program Office of Health Assessment and Translation (OHAT) RoB rating tool (OHAT Risk of Bias Rating Tool for Human and Animal Studies 2015). We performed a pilot test to test the feasibility of using the OHAT RoB tool for included studies. Studies were assigned to one of three tiers based on ratings of key criteria, and other RoB criteria. To be a Tier 1 study (highest quality) all key criteria had to be rated as “definitely low” or “probably low” along with a majority of other RoB criteria receiving these ratings. Tier 3 studies (lowest quality) were defined by “definitely high” or “probably high” ratings of all key criteria accompanied by a majority of other RoB criteria with these ratings. Studies which didn’t fall into either Tier 1 or Tier 3 were assigned as Tier 2. Each criteria rating and tier assignment was discussed in duplicates by SR, RP and MW, disagreements were solved by discussion amongst pairs. Criteria used are described in detail in supplementary materials S1 and supplementary table S3.

### Meta-analysis

Studies were grouped by similar exposure and outcome. For groups with similar outcome, we performed random-effects meta-analyses using the Knapp-Hartung method (Knapp and Hartung 2003) with restricted maximum likelihood estimators. We converted effect estimates to a standardized increment, 1 h for sunshine and 1 W/m^2^ for UV radiation. Relative risks (RR) and 95% confidence intervals were computed. A minimum of four studies with similar or identical exposure and outcome measures and effect estimates that could be pooled (RR, betas, odds ratio, percent change) were required. The median incubation periods for influenza A and B are 1.4 days and 0.6 days respectively (Lessler et al. 2009). Thus, if studies reported more than two lag periods, we took the longer over the shorter period to cover incubation periods and delay in reporting. In addition, lag periods covering several days were preferred to single lags if available. In addition, for sunshine hours when studies provided estimates for extreme low, or extreme high exposure, the extreme low effect size (and its lag) was used for the primary analysis, and extreme high exposure for sensitivity analysis.

I^2^ was used to assess heterogeneity, using the Cochrane handbook’s categories for interpretation (0–40% might not be important, 30–60% may represent moderate heterogeneity, 50–90% may represent substantial heterogeneity and 75–100% may represent considerable heterogeneity) (Deeks et al. 2019). Funnel plots and Egger’s regression test were used to assess publication bias.

### Certainty of evidence

The certainty of evidence was assessed for each exposure-outcome pair using the Grading of Recommendations Assessment, Development and Evaluation (GRADE) approach (Schünemann et al. 2013). Exposure-outcome pairs were assigned an initial rating based on study designs, with observational studies receiving “low” initial ratings. This rating could be reduced by risk of bias, imprecision, inconsistency, indirectness, and publication bias, and increased by the presence of a dose–response gradient, large magnitude of effect and the demonstration of an effect of plausible residual confounding.

### Sensitivity analyses

Sensitivity analyses were conducted (1) by using different methods of random effects estimation (DerSimonian-Laird estimate and the Paule-Mandel estimator), (2) by using extreme high (95% percentile and above) sunshine hours rather than lower percentiles for studies using certain percentiles as comparison rather than a fixed increment, (3) and exclusion of studies with a Tier 3 RoB rating, (4) no given increment, (5) and ILI as outcome. Additionally, univariate meta-regressions were performed on sunshine hours to explore heterogeneity using the R “metafor” package. We included climate region divided into N = 5 categories defined by the Köppen-Geiger classification (Kottek et al. 2006) (Supplementary materials S4, Table S4), virus type (influenza A or B, ILI, or influenza), and lab confirmed vs ICD/symptom confirmed influenza/ILI as moderators if at least three studies were available for the respective moderator. There were not enough studies for a similar analysis for UV radiation available.

Statistical analyses were completed using R version 4.3.2. The study was registered with PROSPERO (Reference number CRD42021248451) and followed the Preferred Reporting Items for Systematic Reviews and Meta-Analyses (PRISMA) (Page et al. 2021), the checklist is provided in supplementary materials S5. This specific review only covers UV radiation and sunshine hours and not all meteorological factors as described in the registration. The complete study protocol is provided in the supplementary materials S1.

## Results

### Description of included studies

**Fig. 1:**
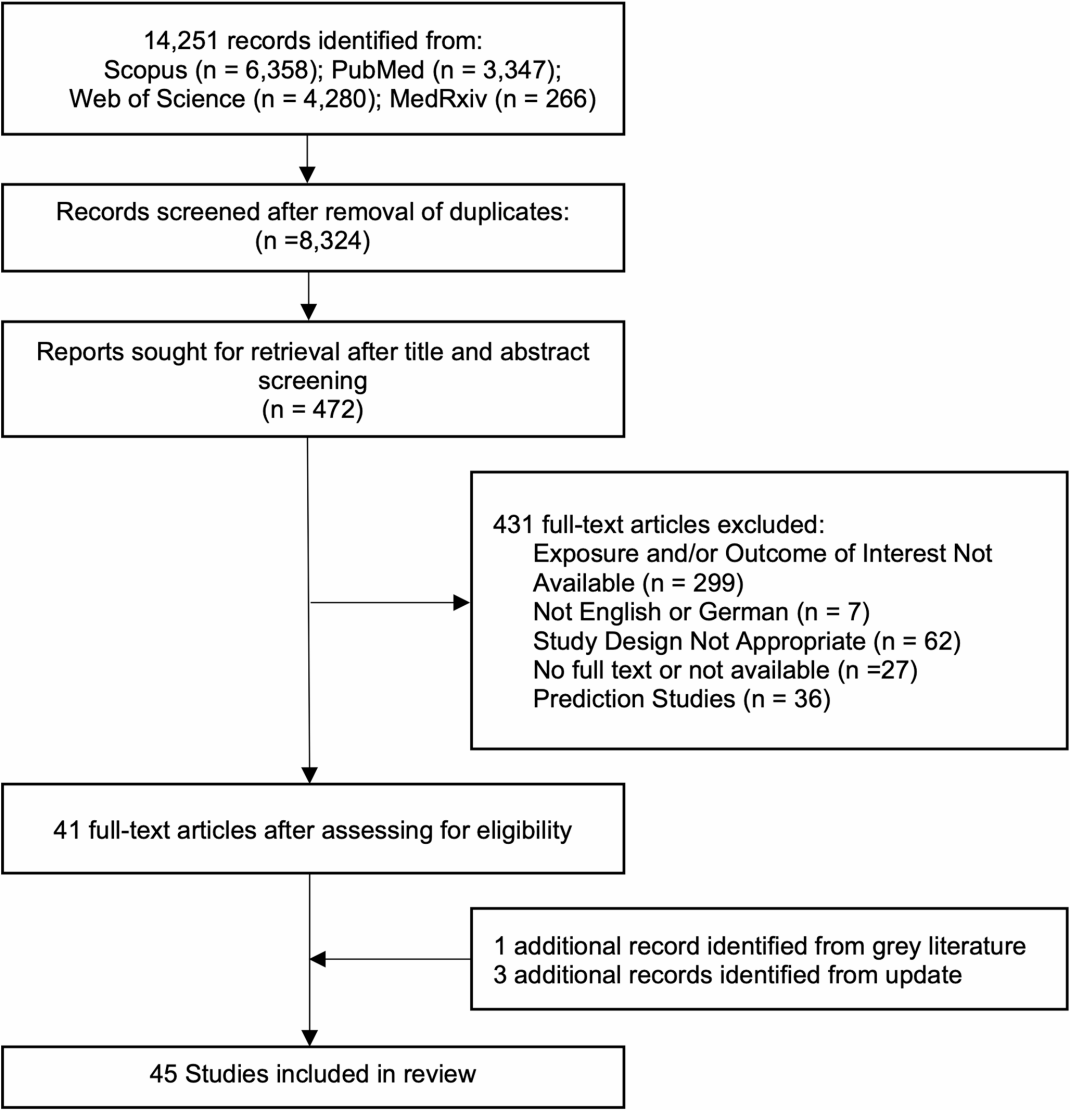
Flowchart of study selection

Our initial search resulted in 14,251 articles and after removal of duplicates and title/abstract screening, 472 full-text articles were assessed for eligibility. We identified 41 full-text articles as eligible for the systematic review (Fig. 1). One additional eligible article was identified from grey literature search and three were identified in the search update. 31 (69%) studies assessed sunshine hours, 12 (27%) studies assessed solar radiation, one study assessed UV index, and one study examined both sunshine duration and solar radiation.

28 (62%) studies were located in Asia, seven (16%) in Europe, four in North America, three included data from multiple continents, two in South America, and one in Oceania. We rated 36 (80%) studies as moderate RoB (Tier 2), five (11%) studies as low RoB (Tier 1) and four (9%) studies as high RoB (Tier 3). A total of 1,314,712 influenza cases were counted across all included studies which provided this information, with 378,960 cases being laboratory confirmed. Further description of all studies can be found in Table 2.

**Table 2:** Study characteristics of all included studies

Study, year	Exposure time frame	Study location	Study period	Virus	*n*	Outcome	Exposure	Direction of effect for studies excluded from meta-analysis	Excluded from meta-analysis; reason	Risk of Bias	Lag^†^
Athanasiou et al. (2023)	Short Term	Europe, Greece	01.01.10—31.12.19	ILI	no info	Incidence	Solar Radiation	n/a	Effect estimates of interest not available	2	–
Chan et al. (2015)	Short Term	Asia, China, Hong Kong	01.01.98—31.12.12	Influenza A, Influenza B	7,655	Hospitalizations	Solar Radiation, Sunshine Hours	n/a	Effect estimates of interest not available	2	–
Charland et al. (2009)	Short Term	North America, USA, 35 cities	01.10.00—30.09.05	Influenza	63,334	Incidence based on Hospitalizations	Solar Radiation	–	–	2	No info
Chattopadhyay et al. (2018)	Short Term	North America, USA, 3,143 Counties	01.01.03—31.12.12	Influenza	no info	Incidence	Solar Radiation	–	–	2	0 weeks **1 week** 2 weeks
Chen et al. (2014)	Short Term	Asia, China, Suzhou	01.01.09–31.12.10	Influenza A	35	Hospitalizations	Sunshine Hours	negative	Effect estimates of interest not available	2	–
Chen et al. (2023)	Short Term	Asia, China, Guangzho	01.01.19–31.12.22	Influenza	210,835	Incidence	Sunshine Hours	–	–	1	0 days 8 days** 9 days* 4 weeks
Chew et al. (1998)	Short Term	Asia, Singapore	01.09.90—01.09.94	Influenza A, Influenza B	430	Incidence based on Hospitalizations	Solar Radiation	n/a	Effect estimates of interest not available	2	–
Chong et al. (2015)	Short Term	Asia, China, Hong Kong	25.08.03—22.08.04	Influenza and Pneumonia	no info	Transmission Rate	Solar Radiation	positive	Insufficient number of studies with same outcome for meta-analysis	2	–
Grant and Giovannucci (2009)	Short Term	North America, USA, 12 US cities	02.12.18—31.12.18	Influenza	42,920	Case Fatality	Solar Radiation	negative	Effect estimates of interest not available	2	–
He et al. (2017)	Short Term	Asia, Japan, Nagasaki Prefecture	03.04.06—07.09.14	Influenza	no info	Incidence	Sunshine Hours	negative	Effect estimates of interest not available	2	–
Ianevski et al. (2019)	Short Term	Europe, Norway, Sweden, Finland, Estonia, Latvia, Lithuania	01.09.10—27.08.17	Influenza	no info	Incidence	UV Index	n/a	Effect estimates of interest not available	2	–
Iha et al. (2012)	Short Term	Asia, Japan, Naha Region	01.01.07—31.07.11	Influenza A, Influenza B	30,603	Incidence	Sunshine Hours	negative, and positive	Effect estimates of interest not available	2	–
Imai et al. (2014)	Short Term	Asia, Bangladesh, Kamalapur	01.01.05—31.12.08	Influenza A, Influenza B	333	Incidence	Sunshine Hours	–	–	2	**0–1** & 0–3 week average
Jing et al. (2020)	Short Term	Asia, China, Gansu Province	01.01.12—31.12.18	Influenza	no info	Incidence	Sunshine Hours	negative	Effect estimates of interest not available	2	–
Jury and Kerr (2022)	Long Term	North America, USA, Puerto Rico North America, USA, Mainland Africa, South Africa	no info	Influenza and Pneumonia	no info	Mortality	Solar Radiation	negative	Effect estimates of interest not available	3	–
Liu et al. (2019)	Short Term	Asia, China, Jiangsu	01.01.11—31.12.16	ILI, Influenza A, Influenza B	16,197	Incidence	Sunshine Hours	negative	Effect estimates of interest not available	2	–
Lu et al. (2021)	Short Term	Oceania, Australia, Brisbane	01.01.15—30.06.19	Influenza	66,971	Incidence	Sunshine Hours	–	–	1	6 weeks* 1 week**
Ma et al. (2022)	Short Term	Asia, China, Shenzhen	01.01.09—31.12.15	Influenza A, Influenza B	3,330	Incidence	Sunshine Hours	negative, and positive	Effect estimates of interest not available	2	–
Martin et al. (1978)	Short Term	Europe, United Kingdom, Newcastle	01.10.71—28.02.77	Influenza A	340	Hospitalizations	Sunshine Hours	negative	Effect estimates of interest not available	2	–
Martins et al. (2020)	Short Term	South America, Brazil, 5 States	01.01.17—31.12.19	Influenza	no info	Incidence based on Hospitalizations	Sunshine Hours	–	–	2	No Info
Ng et al. (2022)	Short Term	Asia, China, Macau	01.01.14—31.12.18	Influenza A, Influenza B	17,104	Incidence based on Hospitalizations	Sunshine Hours	–	–	2	0, **7**, 14, 21 and 27 days
Pan et al. (2019)	Short Term	Asia, China, Panzhihua	01.01.06–31.12.15	Influenza A, Influenza B	737	Incidence	Sunshine Hours	negative, and positive	Effect estimates of interest not available	2	–
Qi et al. (2021)	Short Term	Asia, China, Chongqing	01.01.12–31.12.19	Influenza	7,928	Incidence	Sunshine Hours	–	–	1	**4 weeks**
Rao et al. (2020)	Short Term	Asia, China, Suzhou	01.01.16—31.05.19	Influenza A, Influenza B	672	Incidence	Sunshine Hours	negative, and positive	Effect estimates of interest not available	2	–
Roussel et al. (2016)	Short Term	Europe, France, 11 Regions	01.01.03—31.12.13	Influenza	no info	Transmission Rate, Epidemic Size	Sunshine Hours	n/a	Effect estimates of interest not available	2	–
Shaman et al. (2010)	Short Term	North America, USA, Contiguous US	01.01.72—31.12.02	Influenza and Pneumonia	no info	Mortality Rate	Solar Radiation	positive	Effect estimates of interest not available	3	–
Silva et al. (2014)	Short Term	South America, Brazil, Porto Alegre	01.11.08—31.10.09	ILI	3,698	Incidence based on Hospitalizations	Sunshine Hours	–	–	3	**6 days**
Singh et al. (2021)	Short Term	Asia, India, Varanasi	01.01.17–31.12.19	Influenza and Cold	2,375	Incidence	Solar Radiation	negative	Effect estimates of interest not available	2	–
Soebiyanto et al. (2015)	Short Term	Europe, Germany, Berlin Europe, Slovenia, Ljubliana Europe, Spain, Castila y Leon Asia, Israel, Haifa District Asia, Israel, Central District Asia, Israel, Jerusalem District Asia, Israel, Southern District	01.01.06–31.05.09	Influenza associated ILI/ARI	no info	Incidence	Solar Radiation	–	–	2	**1 week**
Spiga et al. (2016)	Short Term	Europe, France, Rhone Alpes Region	01.01.07–26.02.15	ILI, Influenza	11,389	Hospitalizations	Sunshine Hours	negative	Effect estimates of interest not available	2	–
Su et al. (2020)	Short Term	Asia, China, Jinan City	01.01.13–31.12.16	Influenza A, Influenza B	914	Incidence based on Hospitalizations	Sunshine Hours	negative	Effect estimates of interest not available	2	–
Tamerius et al. (2013)	Short Term	World, 40 Countries	01.01.75–31.12.08	Influenza	no info	Incidence	Solar Radiation	–	–	2	**1 month**
Toczylowski et al. (2021)	Short Term	Europe, Poland, Bialystok	01.01.13 −31.12.19	ILI	345,987	Incidence	Sunshine Hours	–	–	2	**0–1 week average**
Vittecoq et al. (2015)	Short Term	Europe, France, 151 Cities	31.08.09—18.04.10	Influenza A	no info	Incidence	Solar Radiation	–	–	1	**0–1 week average**
Wang et al. (2022)	Short Term	Asia, China, Lanzhou	01.01.10—31.12.19	Influenza	6,701	Incidence	Sunshine Hours	negative	Effect estimates of interest not available	2	–
Xu et al. (2021)	Short Term	Asia, China, 24 Provinces	01.01.09—30.09.13	Influenza	2,665	Incidence	Sunshine Hours	n/a	Effect estimates of interest not available	2	–
Yang et al. (2023)	Long Term	Asia, China, 103 counties in Hubei Province	01.01.17—31.12.19	Influenza	no info	Incidence	Sunshine Hours	negative, and positive	Effect estimates of interest not available	3	–
Youthao et al. (2007)	Short Term	Asia, Thailand, Gulf of Thailand/Andaman Sea	01.01.80—31.12.05	Influenza	no info	Incidence	Sunshine Hours	n/a	Effect estimates of interest not available	2	–
Yu et al. (2013)	Short Term	Asia, China, 88 Cities	01.01.05—31.12.11	ILI, Influenza A, Influenza B	no info	Epidemic Duration, Periodicity	Sunshine Hours	negative, and positive	Two-stage methods	1	–
Zhang et al. (2022)	Short Term	Asia, China, 30 Provinces	01.09.10—31.08.17	Influenza	no info	Reproduction Number	Sunshine Hours	negative	Effect estimates of interest not available. Emailed, no reply	2	–
Zhao et al. (2016)	Short Term	Asia, China, Mainland China	10.05.09—30.04.10	Influenza	127,793	Incidence	Sunshine Hours	n/a	Effect estimates of interest not available	2	–
Zheng et al. (2021)	Short Term	Asia, China, Guangxi	01.01.15—31.10.19	Influenza	93,484	Incidence	Sunshine Hours	negative	Effect estimates of interest not available	2	–
(A) Zhu et al. (2022)	Short Term	Asia, China, 4 Cities	01.01.10—31.12.21	Influenza	45,064	Incidence	Sunshine Hours	–	–	2	0, **3**, 7, or 14 days
(B) Zhu et al. (2024b)	Short Term	Asia, China, Xiamen	01.01.13- 24.01.20	Influenza A	50,278	Incidence	Sunshine Hours	–	–	2	**20 days**
(C) Zhu et al. (2024a)	Short Term	Asia, China, Xiamen	01.01.10–31.12.21	Influenza	21,324	Incidence	Sunshine Hours	–	–	2	**11 days**

*Used for primary analysis as the extreme low sunshine hour exposure

** Used for the extreme high sunshine hour exposure sensitivity analysis

† Lag in **bold** used for analysis

Twentynine (64%) studies were excluded from the meta-analysis and used for the systematic review only: 27 due to lack of effect estimates necessary for meta-analysis, one due to use of two-stage methods (differing from all other studies included) and one due to the use of a method resulting in an effect estimate which could not be pooled with other included studies. For solar radiation, three did not report estimates/directions of relationships, three reported a negative relationship and two reported a positive relationship. For sunshine duration, ten studies reported a negative relationship, five studies reported both positive and negative relationships, for example for different time periods or different influenza subtypes, and six did not report estimates/direction of relationships. The one study using UV index as exposure did not report estimates/directions of relationships.

All studies included for meta-analysis were short-term studies and used incidence or incidence based on hospitalizations. Of the 16 studies included for meta-analysis, eleven (69%) assessed sunshine hours, and five (31%) assessed solar radiation. In all included studies, UV-related exposure was reported either as total solar radiation or as an aggregate UV index; none of the studies provided wavelength-specific UV metrics such as separate measures of UVA, UVB, or UVC. The solar radiation studies included two studies assessing multiple countries, two studies located in the US, and one in France. Only one solar radiation study reported number of influenza cases, 63,334, which were not laboratory confirmed. The sunshine duration studies included six studies located in China, two in Brazil, one in Australia, one in Poland, and one in Bangladesh. Several sunshine duration studies reported the number of influenza cases leading to a total of 769,768 cases, 92,582 of which were laboratory confirmed.

### Quality rating of studies included in the meta-analysis

Four solar radiation studies were rated with moderate RoB (Tier 2) and one was rated low RoB (Tier 1). For the key RoB criteria, all solar radiation studies were rated definitely low RoB due to exposure misclassification. Second, for bias due to confounding four were rated a probably high RoB and one study received probably low RoB. Third, for outcome measurement bias three were rated probably low RoB, one as probably high RoB, and one as definitely low RoB. Additionally, all solar radiation studies were rated probably high RoB for attrition bias.

Seven sunshine duration studies were rated with moderate RoB (Tier 2), three were rated as low RoB (Tier 1) and one was rated as high RoB (Tier 3). As for the key RoB criteria for sunshine duration, nine studies were rated probably high RoB for exposure misclassification and two were rated definitely low RoB. Second, for bias due to confounding seven studies were rated as probably low RoB, three as probably high RoB and one as definitely low RoB. Third, for outcome measurement bias, four studies were rated definitely low RoB, five as probably low RoB, and two as probably high RoB. Notably, for attrition bias, nine studies were rated as probably high RoB, and two studies as definitely low RoB.

Overall quality of evidence for solar radiation and sunshine duration were judged to be “very low”. Full certainty of evidence assessments are available in Table 3. Testing for publication bias using funnel plot asymmetry was not possible for solar radiation because less than 10 studies were available, which would lead to low statistical power (Page et al. 2019). For sunshine duration, the majority of studies fall within the 95% confidence interval of the plot and demonstrate high precision, as indicated by their clustering near the top of the funnel (Supplementary Figure S12). Egger’s regression test revealed no significant asymmetry *p* = 0.56, suggesting the absence of substantial publication bias.

**Table 3:** Description of certainty of evidence ratings for solar radiation and sunshine duration

		**Solar radiation**		**Sunshine duration**
**Quality of evidence**				
**Downgrade**				
	Rating	Rationale	Rating	Rationale
Risk of bias across studies	−1	Overall, 4/5 studies rated (Tier 2) moderate RoB, and one study rated (Tier 1) low RoB. No studies were rated high RoB (Tier 3). Of the three key criteria, bias due to confounding and outcome measurement bias had 4/5 and 1/5 studies, respectively, rated as “probably high” RoB while exposure measurement was rated as “definitely low” RoB for all studies. Additionally, there were several probably high ratings for both attrition bias and selection bias. Because of these serious limitations we downgraded the quality of evidence by one level. All studies were rated as “definitely low” for exposure misclassification and bias due to COI and “probably high” for attrition bias. For bias due to confounding four were rated a probably high RoB and one study received probably low RoB. Regarding outcome measurement bias three were rated probably low RoB, one as probably high RoB, and one as definitely low RoB. For selection bias 3/5 studies were rated “probably high” RoB, and 2/5 “probably low”. For reporting bias, 4/5 studies were rated “definitely low” RoB and 1/5 rated “probably low” RoB. For appropriateness of statistical methods, 3/5 were rated “probably high” RoB, and 2/5 “probably low” RoB	−1	Overall, 7/11 studies were rated with moderate RoB (Tier 2), three were rated as low RoB (Tier 1) and one was rated as high RoB (Tier 3). All three key criteria, exposure misclassification, bias due to confounding, and outcome measurement bias, had studies with “probably high” RoB” ratings with 9/11, 3/11 and 2/11 respectively. Additionally, attrition bias was rated as “probably high” RoB for 9/11 studies. Because of these serious limitations we downgraded the quality of evidence by one level. 9/11 studies were rated probably high RoB for exposure misclassification and 2/11 were rated definitely low RoB. For bias due to confounding 7/11 studies were rated as probably low RoB, 3/11 as probably high RoB and 1/11 as definitely low RoB. For outcome measurement bias, 4/11 studies were rated definitely low RoB, 5/11 as probably low RoB, and 2/11 as probably high RoB. Notably, for attrition bias, 9/11 studies were rated as probably high RoB, and 2/11 study as definitely low RoB. 6/11 studies were rated “definitely low” RoB for selection bias, three were rated “probably low” RoB and two were rated “probably high”. 10/11 studies were rated “definitely low” RoB for reporting bias, and one was rated “probably low” RoB. 9/11 were rated “definitely low” RoB for bias due to statistical methods and 2/11 were rated “probably low” RoB. 10/11 were rated “definitely low” RoB due to COI and one was rated “probably low” RoB
Inconsistency	−1	We downgraded for inconsistency because the I^2^ is considerable at 94.9%	−1	We downgraded for inconsistency because the I^2^ is considerable at 97.0%
Indirectness	0	Influenza cases were identified using direct measures, laboratory confirmation or ICD codes for 4/5 studies, and a direct measure of exposure was used	−1	Influenza cases were identified using direct measures, laboratory confirmation or ICD codes for 911 studies, but sunshine duration is an indirect measure of exposure
Imprecision	0	We judged that the 63,334 events were sufficient for meta-analysis. Additionally, the confidence interval of the pooled estimate was adequately narrow	0	We judged that the 769,768 events were sufficient for meta-analysis. Additionally, the confidence interval of the pooled estimate was adequately narrow
Publication Bias	-	We were unable to assess publication bias because less than 10 studies were meta-analyzed	+ 1	We judged that based on the funnel plot and Egger’s regression test there is no indication of relevant publication bias, so we upgraded
**Upgrade**				
Large Magnitude of Effect	0	We judged that the pooled estimate was not large as it was greater than 0.5, therefore we did not upgrade	0	We judged that the pooled estimate was not large as it was greater than 0.5 and less than 2, therefore we did not upgrade
Dose–Response	+ 1	We upgraded because studies suggested an exposure–response gradient i.e. increase in W/m^2^ solar radiation decreases risk of influenza incidence	0	Studies did not suggest an exposure–response gradient is present, so we did not upgrade the score
Plausible Confounding	0	We judged that many studies may have residual confounding because they did not adjust for all important confounders, so we did not upgrade	0	We judged that many studies may have residual confounding because they did not adjust for all important confounders, so we did not upgrade
**Summary of the score:**				
Overall rating (initial rating is “low”)	Very Low	Low – (2) + (1) = Very Low	Very Low	Low – (2) + (1) = Very Low

### Associations between exposure and outcome

**Fig. 2:**
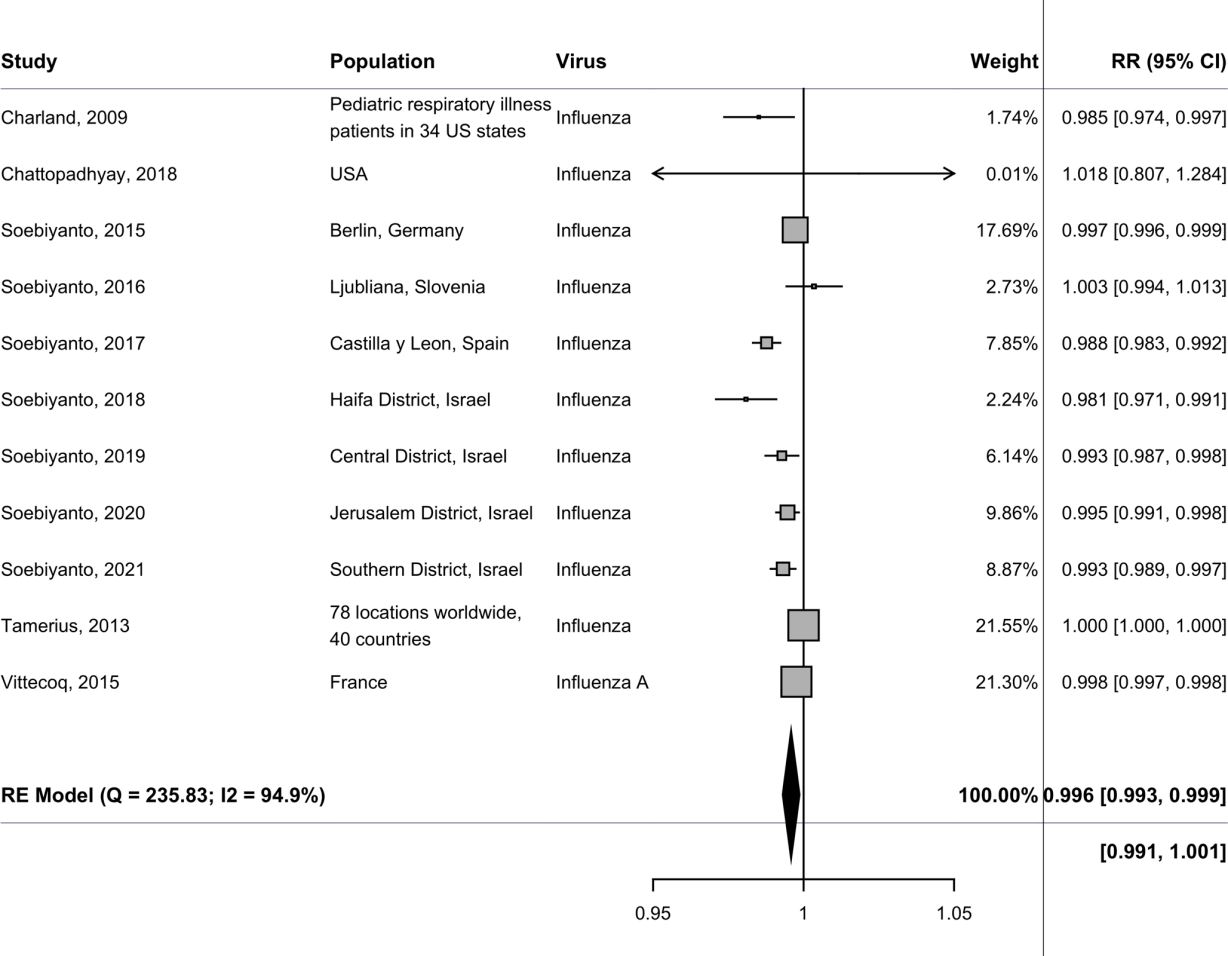
Findings from the random-effects meta-analysis showing relative risks and 95% confidence intervals (RR [95% CI]) for influenza incidence, corresponding to a change per 1 W/m^2^ increase in solar radiation

**Fig. 3:**
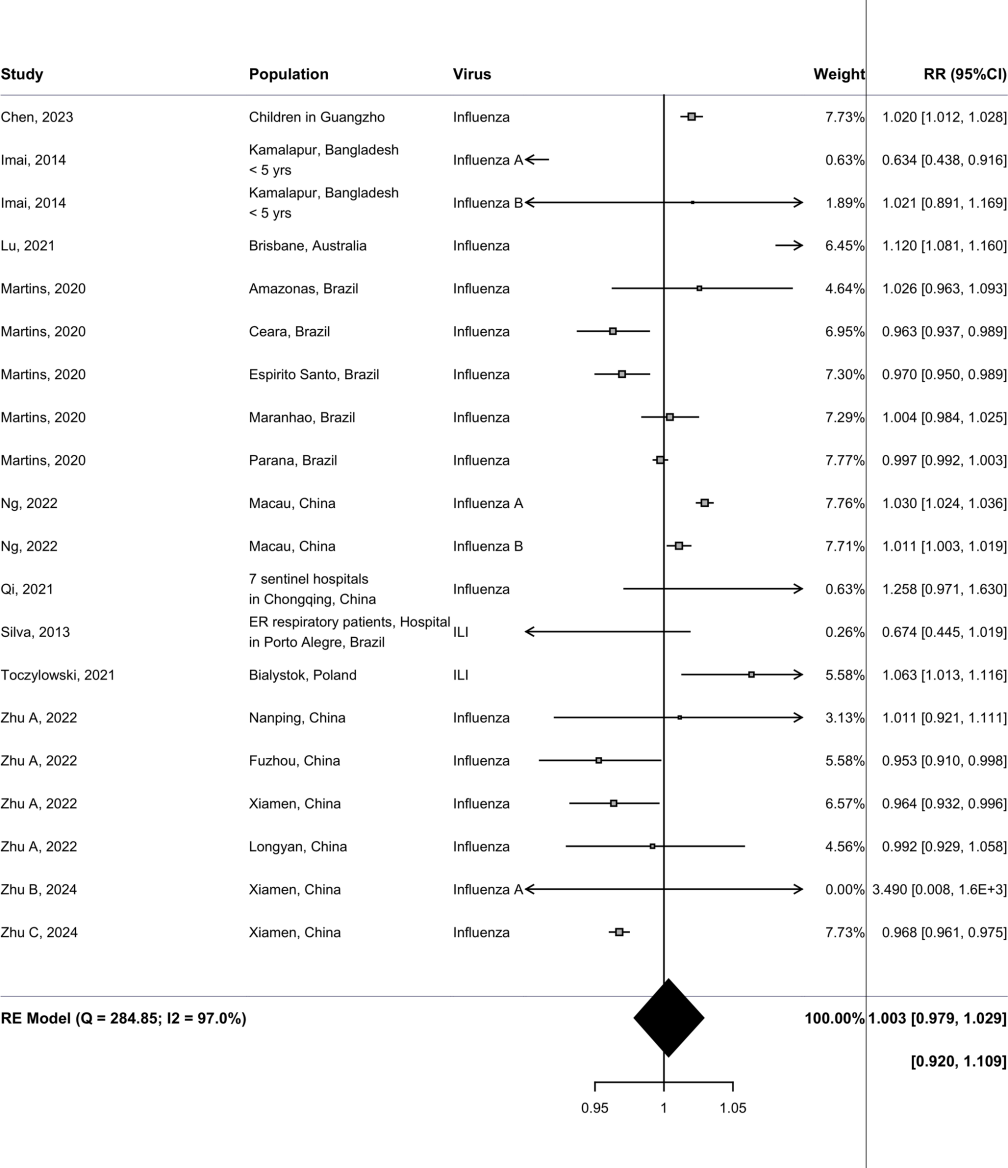
Findings from the random-effects meta-analysis showing relative risks and 95% confidence intervals (RR [95% CI]) for influenza incidence, corresponding to a change per 1 h increase in sunshine duration

Analysis of solar radiation pooled estimates showed that every 1 W/m^2^ increase was significantly associated with a decrease in influenza incidence (RR 0.996 [0.993–0.999]), indicating a small protective effect of solar radiation on influenza incidence, although the heterogeneity was high (I^2^ = 94.9%) (Fig. 2). In the meta-analysis of sunshine duration, pooled estimates showed no significant association between a 1 h increment in sunshine duration and influenza incidence (RR 1.003 [0.979–1.029]) with high heterogeneity (97.0%) (Fig. 3).

**Fig. 4:**
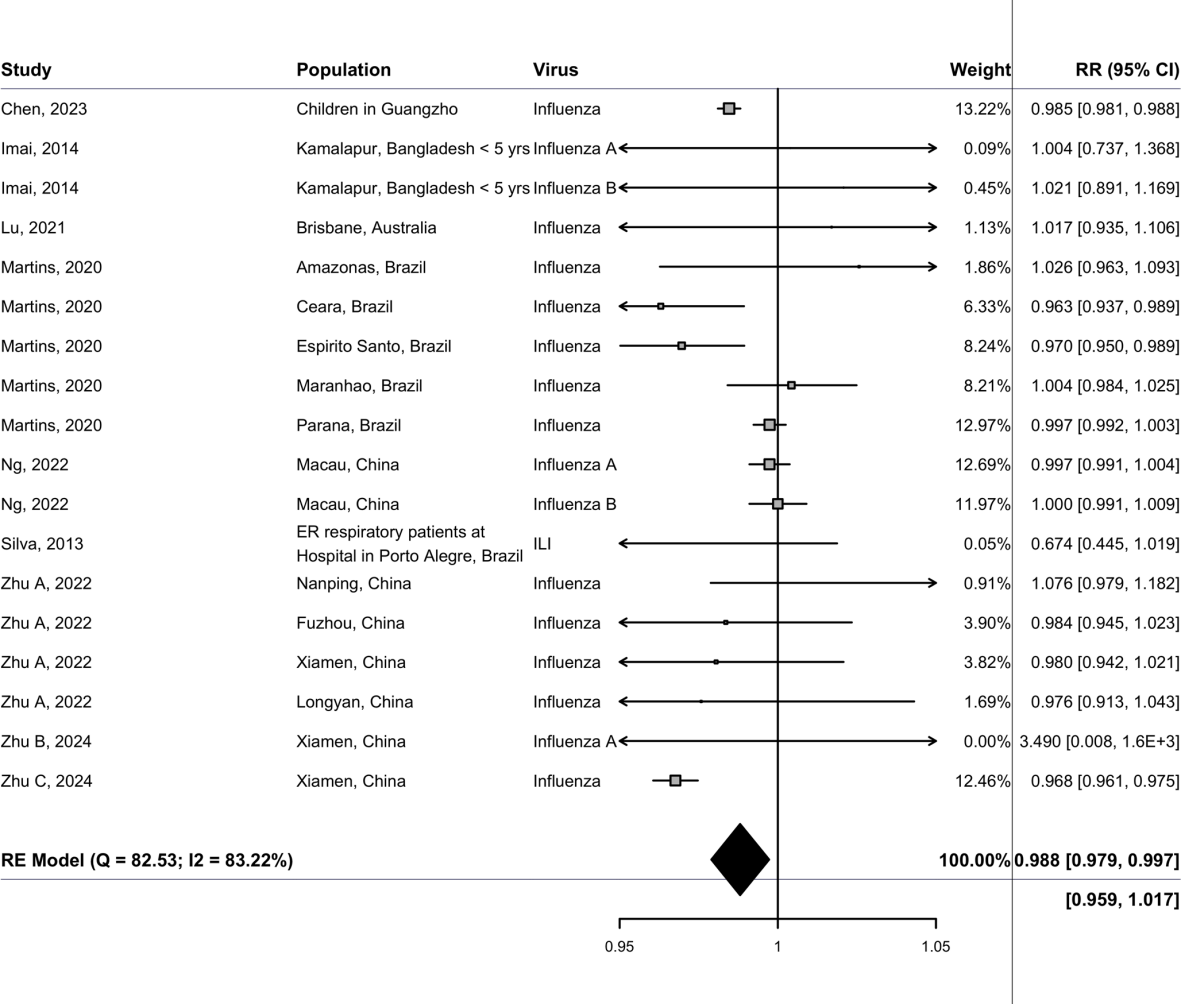
Findings from sensitivity analysis for a random-effects meta-analysis showing relative risks and 95% confidence intervals (RR [95% CI]) for influenza incidence, corresponding to a change per 1 h increase in sunshine duration using extreme high sunshine exposure

Results when using DerSimonian-Laird and Paule-Mandel estimators continued to show a small protective effect for solar radiation, and no significant association for sunshine duration. For extreme high sunshine duration exposure, results showed a small protective effect (RR 0.988 [0.979–0.997]) (Fig. 4). All other sensitivity analyses for both solar radiation and sunshine duration also demonstrated that results were robust. Additional forest plots for sensitivity analyses can be found in supplementary materials S4, figures S3-S11. The univariate meta-regressions of sunshine hours showed that results differed neither for climate zone nor for virus type. There was a small indication for a higher association for studies using lab-confirmed influenza data vs. influenza coded by ICD codes. The results can be found in supplementary materials S4, figure S13.

## Discussion

This systematic review and meta-analysis synthesized evidence on the relationship between UV radiation and sunshine exposure and influenza/ILI. We identified 45 studies for the systematic review with 16 short-term studies meta-analyzed using random effects meta-analysis, including over 1,000,000 influenza cases. Our analyses indicated a small protective effect for exposure to UV radiation, while we saw no significant association for sunshine.

Influenza viruses are single stranded RNA viruses which are transmitted by infectious droplets in the air and by contact with contaminated surfaces (Influenza (seasonal) 2023). UV irradiation introduces RNA damage via photochemical modification, crosslinking, and oxidative damage (Wurtmann and Wolin 2009), with one study showing that single-stranded RNA is damaged at a much higher rate than double-stranded RNA in vitro (Pearson and Johns 1966). In vitro studies of influenza viruses showed a decline in infectivity with exposure to UV radiation (Henle & Henle 1947; Nishisaka-Nonaka et al. 2018; Sutton et al. 2013). Sagripanti and Lytle found a correlation between high solar virucidal radiation and low influenza A prevalence (as well as the inverse) indicating that the inactivating damage induced by UV radiation is important in the spread of influenza (Sagripanti and Lytle 2007). These findings demonstrate that exposure to UV radiation could inactivate influenza virus while aerosolized or on contaminated surfaces, suggesting that increased environmental UV radiation might lead to a decrease in influenza cases.

Additionally, UV radiation plays an important role in the synthesis of Vitamin D, stimulating the production of pre-D3 in the skin (Bikle 2000). With further metabolism, this pre-product becomes 1,25(OH)2D, the hormonal form of Vitamin D, which regulates the proliferation and function of several cell types that mediate the adaptive and innate immune responses and is a key player in host immune defense (Bikle 2000). Several studies show an increase in Vitamin D levels following seasonal ambient UVB increases, while peaks differ for different latitudes and seasons (Webb 2006). These are sufficient for production of Vitamin D (Kasahara et al. 2013; Khanna et al. 2022; Kimlin et al. 2014). Many studies indicate that increased Vitamin D level is protective against influenza (Grant et al. 2020; Moan et al. 2009; Urashima et al. 2010; Zhu et al. 2021).

The protective effect we observed from solar radiation agrees with our hypotheses about Vitamin D and virus inactivation from UV exposure, while the insignificant effect for sunshine duration did not support them. This difference was unforeseen, as we expected to find similar results for both exposures. Since for all analyses the number of studies was limited, this could be explained by differences in exposure measurement, an insufficient number of studies to show the true effect, or differences in the studies themselves. In addition, solar radiation could be a more accurate exposure to measure for this relationship than sunshine duration, as solar radiation is a direct measure of UV while sunshine duration is an indirect measure, i.e. more hours of sunshine do not necessarily equate sufficient UV radiation to stimulate the synthesis of Vitamin D or to inactivate the virus, while more solar radiation would imply more stimulation of synthesis of Vitamin D and virus inactivation. One study found that at latitudes greater than 40 degrees, in some months, Vitamin D synthesis is limited for all skin types because of insufficient UVB exposure from sunlight (Kift and Webb 2024). This also highlights that this difference could be explained by inherent differences in the studies, like study location and period with factors like latitude and season being relevant to the amount of exposure to UV radiation from sunlight.

The results for solar radiation, for example, are driven by mainly three studies, one of which covers 40 cities world wide, one conducted in France and one in Berlin, Germany, while for the studies on sunshine duration, none had a prominent impact.

In temperate regions, influenza infections show a seasonal cycle, characterized by annual influenza epidemics during their respective winter months while in tropical and subtropical regions influenza seasons are more diverse, often peaking in the rainy season or exhibiting a year-round influenza activity without a specific annual pattern (Tamerius et al. 2013).

The study location might therefore also have an impact on the association, as outdoor weather also affects human behaviour. Low temperatures or lack of sun could encourage individuals to stay indoors, leading to a higher likelihood of indoor crowding (Barreca & Shimshack 2012; Peci et al. 2019; Qi et al. 2021). Indoor crowding facilitates viral transmission through the production of contaminated droplets by coughing or sneezing in an infected individual, which spreads through contact in close quarters (Peci et al. 2019). This leads to a greater viral attack rate in human populations, particularly during winter in temperate regions. However, this behavioural response is also suggested in warm seasons in subtropical climates, as individuals could spend more time indoors with air conditioning systems, leading to indoor crowding (Qi et al. 2021). There is no indication of a pattern regarding climatic regions in the results within our analyses. This is also confirmed by the results of our meta-regression. However, while the majority of studies for sunshine durations comes from tropical and subtropical climate, for solar radiation, the study locations are more evenly distributed across the climate regions. This might be another reason for the differing findings between the two exposures.

Additionally, the certainty of evidence was rated as “very low” for solar radiation and sunshine duration implying that the evidence of the observed associations is very uncertain. It is important to note that observational studies, which are very common in environmental epidemiology, always start with a “low” rating with the GRADE certainty of evidence assessment (Schünemann et al. 2013). However, randomized trials, which start with a “high” rating, cannot be performed on environmental exposures due to practical or ethical reasons. The low rating could hence be misleading due to the automatic downgrade based on use of the preferred study design in this field, and the evidence could have higher certainty than implied by this tool.

Other systematic reviews have also assessed the relationship between respiratory diseases and exposure to UV radiation/sunshine duration and report negative or inconsistent results. A systematic review on Legionnaire’s disease suggested that UV radiation and sunshine duration have a negative association with the sporadic occurrence of the disease (Pampaka et al. 2022). As for COVID-19, Moazeni et al. found contradictory results for both sunshine duration and UV radiation, with studies reporting no association, negative and positive relationships (Malihe Moazeni and Ebrahimi 2023). In another study evaluating Respiratory Syncytial Virus results were inconsistent for both UV radiation and sunshine hours (Tang and Loh 2014). A recent systematic review on the influence of weather and urban environment characteristics on upper respiratory tract infections only included three studies with UV radiation as exposure showing weak and inconsistent associations with respiratory tract infections (Hyrkas-Palmu et al. 2025).

The protective effect we found for UV radiation suggests that it could be used to predict influenza incidence. During the process of sorting and selecting papers for this review, we found many publications which were using UV radiation and/or sunshine duration to make predictions about influenza. We did not include these publications in this systematic review, unless they also provided estimates which used real-life data that fit our eligibility criteria and PECO.

Our results also suggest that exposure to UV radiation might be used as a measure against influenza epidemics. However, in many countries, influenza cases are the highest in months with least natural UV light. Although our results show only a small protective association, on a population level, even a small decrease in the number of influenza cases leads to large decrease in the public health burden, since seasonal epidemics of influenza are estimated to infect about 1 billion people per year (Influenza (seasonal) 2023).

However, it is important to note that the conclusions of this manuscript are drawn from aggregated data and refer to a reduction in influenza on a population level. Therefore, they cannot not and should not be used to guide interventions for individuals, such as targeted artificial UV exposure or the supplementation of Vitamin D.

### Strengths and limitations

This systematic review used a sensitive search strategy with no limits on publication start date and included a diverse set of populations, covering several geographic areas. More than one million cases were included, with approximately 400,000 laboratory confirmed cases. We performed several sensitivity analyses which led to robust results. We provide all information designated in the PRISMA checklist, including codes and excel spreadsheets to allow for replicability.

However, several limitations should be considered. First, the measured UV radiation/sunshine duration does not reflect the actual exposure of individuals. For estimating the actual exposure, one would have to consider human behaviors like sun avoidance on hot days or equip people with UV measurement devices, which is not feasible in this kind of studies. Second, there is no gold-standard RoB tool available to assess observational studies, thus a modified version of the OHAT tool was used which may not provide an accurate assessment of this risk. Third, most of the studies were assigned a RoB rating of 2, suggesting that the overall quality of the studies was only moderate. Fourth, heterogeneity was high for all effect estimates. Fifth, many studies would have been eligible for meta-analysis but did not report effect estimates, only providing exposure–response curves. Sixth, many studies did not provide sufficient information about missing values and data collection, particularly for the exposure measurement. Seventh, many studies reported only one lag, so we were not always able to choose the preferred lag.

## Future recommendations

We identified some key recommendations which future studies should consider. We recommend that detailed information about data collection, including information on data completeness and missing data, and exposure measurement is provided in publications. This includes designating if a model or a monitor was used, the precision of the assigned exposure (i.e. from average from a single monitor, or multiple, closest to address), and providing grid size if using a model. We suggest that authors consider several lags when analysing short-term exposures and provide effect estimates and biological hypotheses for all lags analysed. Important time-varying confounders, including but not limited to season, latitude, day of the week, and time trend, temperature and holiday, should be carefully considered and potentially adjusted for when using a time series design (Krishnan Bhaskaran et al. 2013). Finally, further evidence on this topic needs to be collected to better understand the relationship between UV radiation/sunshine duration and influenza.

## Conclusion

In conclusion, this study provides evidence for a small negative association between exposure to UV radiation duration and influenza. Findings indicate that UV radiation could be used to predict influenza incidence, specifically if additional meteorological variables and their interrelationship with UV radiation are being taken into account. Since causality for the exposure–response association cannot be established from these studies, the question whether UV exposure—through natural or artificial UV light—could be recommended as a preventive measure against influenza warrants further research.

## Supplementary Information

Below is the link to the electronic supplementary material.

Supplementary file1 (DOCX 49782 KB)

## Data Availability

The completed excel files containing all collected data can be made available upon request.
