# Supplementary material for: Associations between sunshine and influenza or influenza-like illness, a systematic review and meta-analysis
Source: Int J Biometeorol. 2026 Jan 16;70(2):29. doi: 10.1007/s00484-025-03121-0 (PMC12811287; doi:10.1007/s00484-025-03121-0)
Supplement: Supplementary file 1 — Supplementary file1 (DOCX 49782 KB) [file 484_2025_3121_MOESM1_ESM.docx]

**SUPPLEMENTARY MATERIALS**

**Associations between sunshine and Influenza or Influenza-like illness, a systematic review and meta-analysis**

Savanna Ratky, Rui Cheng Javier Chai, Alexandra Schneider, Susanne Breitner-Busch, Annette Peters, Margarethe Woeckel, Regina Pickford.

**Supplementary Materials S1 – Study Protocol**

**Introduction**

Influenza and influenza-like illness (ILI) are common acute respiratory diseases which contribute an immense burden to public health globally. Infections have been observed to increase in winter in temperate regions, and generally increase in rainy seasons in tropical regions (Charland et al., 2009; Roussel et al., 2016). This observation suggests that the environment could play a role in influenza and ILI activity. Thus, there is growing academic interest in investigating the environmental risk factors of influenza and ILI.

Description of exposures

This systematic review will focus on solar radiation, ultraviolet (UV) radiation and sunshine duration. UV radiation has been demonstrated to have a virucidal effect through the inactivation of viral particles in the environment (Sagripanti & Lytle, 2007). Additionally, exposure to UV radiation is linked to increased human immunity through the production of vitamin D. This reduces an individual’s susceptibility to influenza infections, as vitamin D plays a role in modulating the human innate and adaptive immune systems (Aranow, 2011; Peci et al., 2019; Roussel et al., 2016). Such an effect has been shown in several studies where solar UV radiation is negatively correlated with influenza incidences. For instance, the lack of solar UV radiation during winter in temperate regions is linked to greater influenza incidences (Peci et al., 2019; Roussel et al., 2016).

Description of outcome

Influenza is a common acute respiratory disease caused by the influenza virus. While most influenza cases are caused by the influenza A virus (IAV), influenza B virus and rarely, influenza C virus, can also cause influenza in humans (Lindner-Cendrowska & Bröde, 2021; Yan et al., 2024). Laboratory diagnosis typically uses an antigen test or reverse transcription polymerase chain reaction (Stellrecht, 2017).

Influenza morbidity exhibits a seasonal variation most notable in temperate climates, with epidemics occurring in late autumn to early spring, and peaking during winter (Feng et al., 2016; Lindner-Cendrowska & Bröde, 2021). Influenza has been attributed to a substantial proportion of excess mortality in the winter as well (Nielsen et al., 2011). This variation is associated with the rainy season in subtropical to tropical climates (Dave & Lee, 2019; Suntronwong et al., 2020; Zhang et al., 2022). The magnitude of epidemics varies by year, population, and location (Lindner-Cendrowska & Bröde, 2021).

In rare cases, IAVs of avian or swine origin can infect humans. Such transmission routes include prolonged, close, and unprotected contact with infected avian or swine fomites (Kandun et al., 2006). Most of these infections may be driven by the poultry and swine industries, where proximate contact with infected poultry by workers is extremely likely (Du Ry van Beest Holle et al., 2005). As such infections usually occur in occupational settings, these viruses were excluded for this systematic review.

ILI, however, is defined by symptoms, such as a fever greater than or equal to 38 °C and cough or sore throat without an alternative cause (Toczylowski et al., 2021). Other notable symptoms include malaise, and nausea, which occur during immune system activation, when cytokines are released (Stellrecht, 2017). While various pathogens can cause ILI, influenza viruses alone account for as much as 35 – 45% of ILI cases during peak seasons (Stellrecht, 2017). Other pathogens include respiratory syncytial viruses, rhinoviruses, enteroviruses, human coronaviruses, and adenoviruses (Spencer et al., 2022). Although most infections are self-limiting, influenza and ILI greatly burden public health, with consequences on morbidity, mortality, and economic burdens.

**Supplementary Figure S1**: Directed acyclic graph of relationship between meteorological factors and influenza and influenza-like illness.


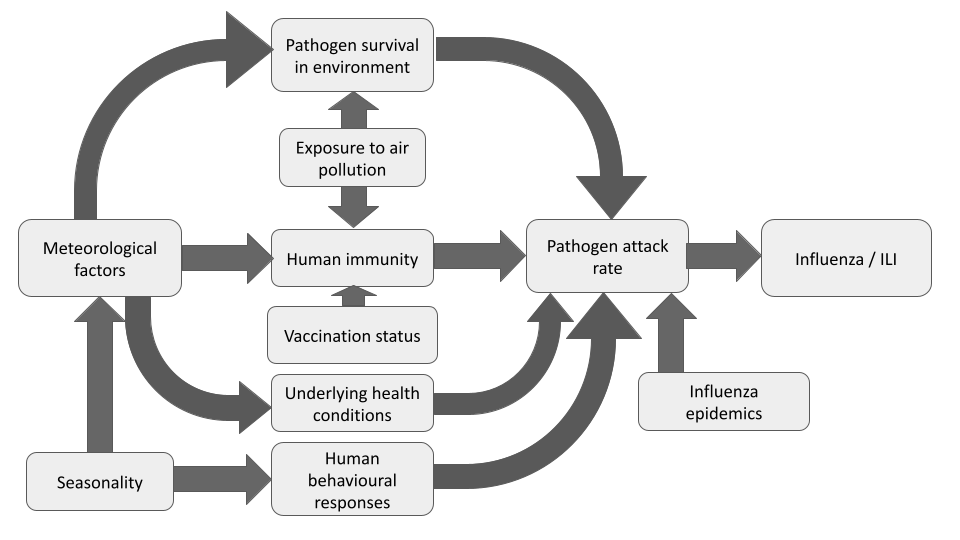


Two critical confounders are shown, but demographic factors such as age, sex, ethnicity, income, marital status, and socioeconomic status may be potential confounders as well.

Confounders

Confounders are variables that influence both exposures and outcomes, forming a spurious correlation between exposure and outcome. This introduces a bias by confounding, posing a threat to a study’s internal validity (Frampton et al., 2022). There are several critical and potential confounders that could complicate discerning the effects of a specific meteorological factor on influenza and ILI, where two critical ones are illustrated in the directed acyclic graph (DAG) in Figure S1.

People with chronic diseases such as type 2 diabetes mellitus could be more vulnerable to influenza and ILI infections, as chronic diseases impair the ability of the immune system to combat infections (Walker et al., 2020; Zhong et al., 2015). For instance, Zhong et al. (2015) found that individuals with type 2 diabetes mellitus and liver disease had lower salivary terminal α2-3-linked sialic acids than healthy individuals, which is a biomarker used to assess susceptibility to avian influenza infection. Walker et al. (2020) also found that the symptoms of chronic diseases may be exacerbated by influenza infection, leading to a greater risk of severe disease. A proposed example is the induction of inflammatory responses by influenza viruses, leading to endothelial dysfunction, which could aggravate atherosclerosis (Fagnoul et al., 2013; Walker et al., 2020). When compounded with exposure to sub-optimal ambient temperatures, which have been associated with greater morbidity in individuals with chronic diseases, an individual’s susceptibility to influenza and ILI may be increased.

Seasonality plays a huge role in influencing meteorological factors across climate zones. Although the tropics exhibit less pronounced seasonality in temperature and UV radiation, precipitation is generally higher during rainy seasons, and lower during dry seasons. Temperate climates typically exhibit greater seasonality in UV radiation, being higher in the summer and lower in the winter. Hence, seasonality is identified as a critical confounder when investigating the relationship between UV radiation and influenza and ILI.

Another confounder is ozone. It has been proposed to have a virucidal effect, as viruses with a lipid membrane are most sensitive to that air pollutant (Alimohammadi & Naderi, 2021). However, in vitro experiments exposed viral particles to ozone concentrations substantially higher than those in ambient air (Alimohammadi & Naderi, 2021; Tseng & Li, 2008). Hence, the virucidal property of ozone in ambient air is unclear. Potential confounders may include demographic factors including age, sex, and socioeconomic status, which can affect where individuals may live, or how individuals respond to climatic changes (Hossain et al., 2019).

Rationale for the systematic review

Under climate change, there are concerns that disease trends of influenza and ILI could also be altered by global warming and altered weather patterns (IPCC, 2023). He et al. (2023) suggested that respiratory infections like these are driven by at least a meteorological factor, categorising them as ‘climate-sensitive diseases’ (He et al., 2023). However, there are conflicting results reported regarding the presence of an association between meteorological factors, specifically UV radiation and influenza transmissibility or activity (Park et al., 2020; Yan et al., 2024).

This necessitates a systematic review of currently available literature, to synthesise such findings in a meta-analysis, and identify knowledge gaps where present.

**Methods**

Study Objectives

This study provides a study protocol for the evaluation of the effects of exposure to a 1 hour increase of sunshine duration, and/or a 1 unit increase in UV index, and/or a 1 MJ/m2 or W/m² increase in UV radiation, and/or a 1 MJ/m2 or W/m² increase in solar radiation on influenza or influenza-like illness in human populations, through a systematic review and meta-analysis of observational studies.

Solar radiation, measured as W/m^2^, refers to all electromagnetic energy emitted by the sun, including visible, infrared and UV wavelengths (NASA, 2025).

The development of the systematic review protocol was guided by the recommendations from the Conduct of Systematic Reviews in Toxicology and Environmental Health (COSTER). Planning and conduct of the systematic review will also follow COSTER recommendations (Whaley et al., 2020). This protocol follows the guidelines of the Preferred Reporting Items for Systematic Reviews and Meta-Analysis Protocols statement (PRISMA-P). This protocol is registered in the International Prospective Register of Systematic Reviews (PROSPERO) under the reference number CRD42021248451.

PECO Statement and PECOS Statements for Eligibility

This systematic review aims to answer the following question: “In the general human population, what association does an incremental effect of a 1 unit increase in exposure to UV radiation, sunshine or UV index, have with the incidence, mortality, severity and clinical course of influenza/influenza-like illness?”

Eligibility criteria

Eligible studies will be in English or German, with available full texts. They will use human populations as study populations.

Eligible studies will assess influenza or ILI incidence, course, severity, or mortality as outcomes. Derivation of effect measures would vary by study design, resulting in various effect measures including relative risks, hazard ratios, incidence rate ratios, and odds ratios. Cases may be defined as patients with a positive antigen or viral test, patients diagnosed by a physician or an influenza registry as positive, or patients whose cause of death is certified to be virus-related. ILI cases will be defined by symptoms. Furthermore, qualitative research and studies that do not present original data will be excluded.

Eligible studies for meta-analysis will report risk estimates such as relative risk with an increment in the exposures.

A full codified list of eligibility criteria is provided in Table S2. This coding will be used to inform the rationale for the exclusion of ineligible studies in the screening and study selection phases. Some eligibility criteria are not codified as these could be identified and excluded during the title and abstract screening phase.

Literature search

Two search strategies will be employed for observational or human epidemiological studies. The first strategy involves a comprehensive search of scientific literature compiled in databases. The main databases to be searched are PubMed, Scopus, Web of Science, and the preprint server MedRxiv. Search terms will need to be adjusted depending on the database.

Grey literature is defined as ‘that which is produced on all levels of government, academics, business and industry in print and electronic formats, but which is not controlled by commercial publishers’ (Godin et al., 2015; Paez, 2017; Pappas & Williams, 2011). These include academic papers, dissertations, reports, and conference transcripts, but are not typically published in academic sources like peer-reviewed journals. Grey literature formats to be searched are technical reports, white papers, and governmental research.

Two grey literature search strategies will be used. The first strategy will involve searching grey literature databases and catalogs that may contain literature relevant to the review subject. These include Grey Matters, a checklist developed by the Canadian Agency for Drugs and Technologies in Health (CADTH) that provides a structured approach to identifying health-related grey literature from health technology assessment agencies, government organizations, and clinical guideline producers. In addition, WorldCat, a global catalog of library collections, will be searched to identify relevant materials such as books, dissertations, conference proceedings, and technical reports held in libraries worldwide.

The second search strategy will involve searching targeted websites of relevant public health organisations and environmental agencies. This may be guided by the CADTH tool Grey Matters, which offers practical tips on navigating websites commonly used in systematic reviews and ensures comprehensive coverage of grey literature sources (CADTH, 2025). Additionally, technical reports, white papers, and unpublished governmental research will be searched from institutions including the World Health Organization (WHO) and the Environmental Protection Agency (EPA). For both grey literature search strategies, search terms from Supplementary Materials S2 will be used, although the syntax of the search string may be adjusted depending on the database or targeted website.

We will not put any limit on the publication period. The search will be rerun shortly before the final analyses. Duplicate studies yielded from database searches will be identified and removed prior to study selection. However, multiple reports of the same research will not be excluded before study selection.

Study selection

Firstly, two reviewers will screen articles for the title and abstract, with those irrelevant to the PECOS statements defined being designated for exclusion results compared. If there is uncertainty in evaluating a study’s relevance, the study will be included for full text screening. Studies will only be excluded if both reviewers designate the studies for exclusion. Disagreements can be resolved through discussion. In case of no consensus, a third independent reviewer will assist in resolving the conflict.

Full text screening will then be conducted by two reviewers for articles that fulfill the title and abstract screening eligibility criteria. Articles failing to meet the eligibility criteria in the full text screening will be excluded from the study. Reasons for exclusion will be codified, such as “E1 – Full text does not exist for the respective study” (Table S1). Disagreements regarding exclusion criteria or reasons for exclusion will be resolved by discussion, otherwise, a third independent reviewer will be consulted. If a study is classified to be excluded during full text screening, the exclusion criterion with a lower code number precedes the reason for exclusion (Table S1). The screening process and inclusions will be visualised using a PRISMA flowchart.

**Supplementary Table S1**. Codified reasons for exclusion to be used for studies designated for exclusion during full text screening.

| Code | Reason for exclusion |
| --- | --- |
| E1 | Full text does not exist for the respective study. |
| E2 | Study does not use a human epidemiological study design. |
| E3 | Study does not focus on the defined exposures of interest. |
| E4 | Study does not focus on the defined outcomes of interest. |

Data extraction

The features of an included study that will be extracted comprise information on the reference, basic information, exposure, statistics, confounders and outcome. A full list of data fields to be extracted from included studies is found in Table S2.

These data fields will be filled in by two reviewers independently in an Excel spreadsheet, and the results are then compared. Disagreements will be resolved by discussion, and if this is not possible, by the opinion of a third reviewer. If complete descriptions of crucial data fields are missing, such as exposure and outcome assessment, and risk estimates, individual authors will be contacted and asked for the missing information.

Standardisation of risk estimates will be conducted, such that risk estimates are scaled to 1 h increment in sunshine duration, 1 W /m² increase in UV index and 1 MJ/m^2^ increase in solar radiation.

**Supplementary Table S2**. Types of data to be extracted from included studies, sorted by category.

| Reference details | Authors | Journal including DOI |
| --- | --- | --- |
|  | Publication year |  |
| Basic information | Study design | Study period |
|  | Pooled analysis | Continent |
|  | Study population | Country |
|  | Number of events | City / Region |
|  | Population selection | Peer reviewed |
|  | Language | Time frame |
| Exposure | Specific description of exposure | Exposure assessment (model/monitor) |
| Exposure monitors | Completeness of data | Handling of missing data |
|  | Assigned exposure |  |
| Exposure models | Quality assessment | Time-varying exposure |
|  | Model Precision | Assigned exposure |
|  | Grid size |  |
| Exposure measures | Exposure measure | Exposure description |
|  | Unit of exposure |  |
| Statistics | Statistical method | Lag(s) |
|  | Method for confounder selection |  |
| Short-term confounders | Seasonality | Population density |
|  | Latitude/Longitude/Climate zone | Age |
|  | Day of the week | Comorbidity |
|  | Time trend | Other confounders |
|  | Holiday | Vaccination status |
| Long-term confounders | Latitude/Longitude/Climate zone | Population density |
|  | Age | Comorbidity |
|  | Vaccination status |  |
| Outcome | Virus | Outcome |
|  | Virus captured by | Outcome source |
|  | Estimate type | Increment |

Internal validity evaluation

Internal validity refers to the extent to which a cause-effect relationship is supported and established in a piece of evidence or an individual study, that other factors cannot explain (Rooney et al., 2016). This is often done by risk of bias (RoB) assessment for each included study in the systematic review.

The Office of Health Assessment and Translation (OHAT) risk of bias rating tool by the United States’ National Toxicology Program (NTP) was selected as the most favourable tool to evaluate internal validity (NTP, 2015). The risk of bias tool assesses potential sources of bias through 10 primary guiding questions at an outcome level, six of which apply to cohort and case-control study designs. These domains are, bias due to confounding, selection bias, bias due to exposure misclassification, attrition bias, measurement bias, and reporting bias. An additional “other” domain is also available, which includes brief guidance on evaluating other sources of bias such as appropriateness of statistical methods. A four-point scale functions as the answer format for each guiding question, ranging from “definitely low” and “probably low”, to “probably high” and “definitely high” (Figure S2). Should insufficient information be available to make a judgement, “not reported” or “NR” is used instead. Detailed criteria are provided for each guiding question except the “other” domain, for each study design the tool was developed for. Additionally, where possible, considering the direction and magnitude of potential bias is encouraged, but not compulsory (NTP, 2015). Table S3 shows the risk of bias criteria developed for these analyses.

Study-level RoB may be assigned by a three-tier system, with a study with the highest quality rated as “Tier 1”, and the lowest quality rated as “Tier 3” (NTP, 2019). Key RoB criteria and other RoB criteria have to be predefined to assign a rating. For instance, key criteria can include bias due to confounding, bias from exposure assessment, and bias from outcome measurement, while other relevant domains are grouped under “other RoB criteria”. For a study to be rated as “Tier 1”, it has to receive “definitely low” or “probably low” ratings for key criteria, and a simple majority of other RoB criteria to receive “definitely low” or “probably low” ratings. Similarly, for a study to be rated as “Tier 3”, it has to receive “definitely high” or “probably high” ratings for key criteria, and a simple majority of other RoB criteria to receive “definitely high” or “probably high” ratings. Studies failing to meet Tier 1 and Tier 3 criteria will be designated Tier 2 studies (Figure S2) (NTP, 2019). An advantage of this method is that certain RoB domains can be weighted more than others, as some sources of bias could be deemed more threatening to a study’s quality than others. As such, domains defined as “key criteria” are weighted slightly more than the other criteria, but each domain is given equal weighting within the respective groupings. However, precaution must be exercised when deciding if a poor-quality study should be excluded from further analysis, instead, sensitivity analyses will be conducted to evaluate the net effect of such biases (Eick et al., 2020; Steenland et al., 2020).

**Supplementary Table S3**. Risk of Bias Criteria Details

|  | Definitely low | Probably low | Probably High | Definitely High |
| --- | --- | --- | --- | --- |
| Bias due to confounding | Many important confounders adjusted for from one or more confounder blocks | 1-3 important confounders adjusted for from one or more confounder blocks | No important confounders adjusted for but no evidence of confounding demonstrated OR no information provided about confounders | Confounding demonstrated but not adjusted for appropriately |
| Bias due to exposure misclassification | Using a model with information about grid size, OR information about location of monitors with sufficient information about completeness of data and missings | Exposure consistently assessed i.e. using a model (without information about missings/grid size), OR using monitors with some information about either missings/completeness or locations/amount of monitors | Insufficient information provided about how exposure was collected i.e. no information about missings/completeness, location, amount of monitors, precision etc. | Exposure measured using methods with poor validity OR evidence of exposure misclassification present |
| Bias due to outcome measurement | Using laboratory confirmed data | Using ICD codes | Using a combined outcome measure i.e. influenza and pneumonia OR insufficient information provided about outcome measurement OR using symptoms or survey/questionnaire data asking about patient symptoms for which there is indirect evidence that misclassification is present | Using insensitive methods like symptoms or survey/ questionnaire data asking about patient symptoms for which there is direct evidence that misclassification is present |
| Selection Bias | Direct evidence that cases were selected using a method /from sites that are representative of study population | Indirect evidence that cases were selected using methods/from sites which may be missing some cases in the study population i.e. only collecting data from 3 hospitals in a large city OR no specific information about how many sites/where data is collected from in study locations but no reason to judge that it is not representative | Indirect evidence that cases selected using methods/from sites which are not representative of study population OR insufficient information provided about how/where cases were collected | Direct evidence that cases were selected using a method /from sites that are not representative of study population |
| Attrition Bias | Direct evidence that missings and exclusions were adequately addressed and documented | Indirect evidence that missings and exclusions were adequately addressed and documented | Not enough information provided about missing data OR evidence that missings or exclusions were large and not adequately addressed | Exclusions not adequately addressed – reasons likely to be related to the true outcome |
| Reporting Bias | All relevant estimates for associations of interest have been reported | Only some relevant estimates are reported but some correlations, sensitivity and other secondary analyses not reported | Most relevant estimates not reported OR insufficient information provided | Measured estimates not reported |
| Bias due to appropriateness of statistical methods | Using appropriate models with sensitivity analyses such as distributed lag non linear models or generalized additive model AND/OR using appropriate models and assessing multiple lags | Using appropriate models without assessing for multiple lags or performing sensitivity analyses | Using methods which may not appropriately assess the relationship i.e. Spearman/Pearson’s correlation | Using inappropriate methods i.e. regression which does not fit data |
| Bias due to conflicts of interest (COI) | Provide a COI statement stating no COI | No COI statement provided, but no reason to assume COI were present OR COI statement provided citing a COI which should not appreciably increase bias | No COI statement provided, with reason to assume COI were present i.e. funding source OR COI statement provided with COI that could appreciably increase bias | COI statement provided with COI that would certainly increase bias |

Crucial confounders have been identified from the DAG linking meteorological factors and influenza or ILI (Figure S1) and can be used to facilitate RoB assessments for one of the domains in the OHAT Risk of Bias tool. These are seasonality, but also include vaccination status and demographic factors including age, sex, ethnicity, income, marital status, and socioeconomic status.

In assessing the appropriateness of statistical methods, the establishment of lag periods must be considered in included studies. Exposure to meteorological changes or fluctuations has acute and chronic pathophysiological effects in the human airway and immune system, leading to a lag period to allow individuals and pathogens to respond to these fluctuations. Studies measuring short-term exposures define this lag period on a scale of days to weeks, which should be factored into their statistical methods (Tamerius et al., 2013; Yan et al., 2024). Hence, the choice of the lag period needs to be considered. If associations for several lag periods are given, the one which makes the most pathophysiological sense will be selected for the meta-analysis. Another statistical aspect to be considered is the appropriateness of the methods used to control for confounding (NTP, 2015).

Funding sources and conflicts of interest (COI) should be identified during the RoB rating and integrated as a factor when evaluating the risk of bias in selective reporting during internal validity assessment (NTP, 2019), and at other points of the evaluation process (Viswanathan et al., 2012). A limitation to this is the change in reporting standards over time, as requirements for declaring COI and funding sources by journals are a more recent addition.

RoB for included studies will be visualised using a traffic light plot (Figure S2). Internal validity assessment will be conducted by experienced and senior scientists. Justification for rating given to each domain in the RoB tool will be transparently reported.

**Supplementary Figure S2**. An example of a visual summary of internal validity ratings using the OHAT Risk of Bias rating tool for human observational studies.


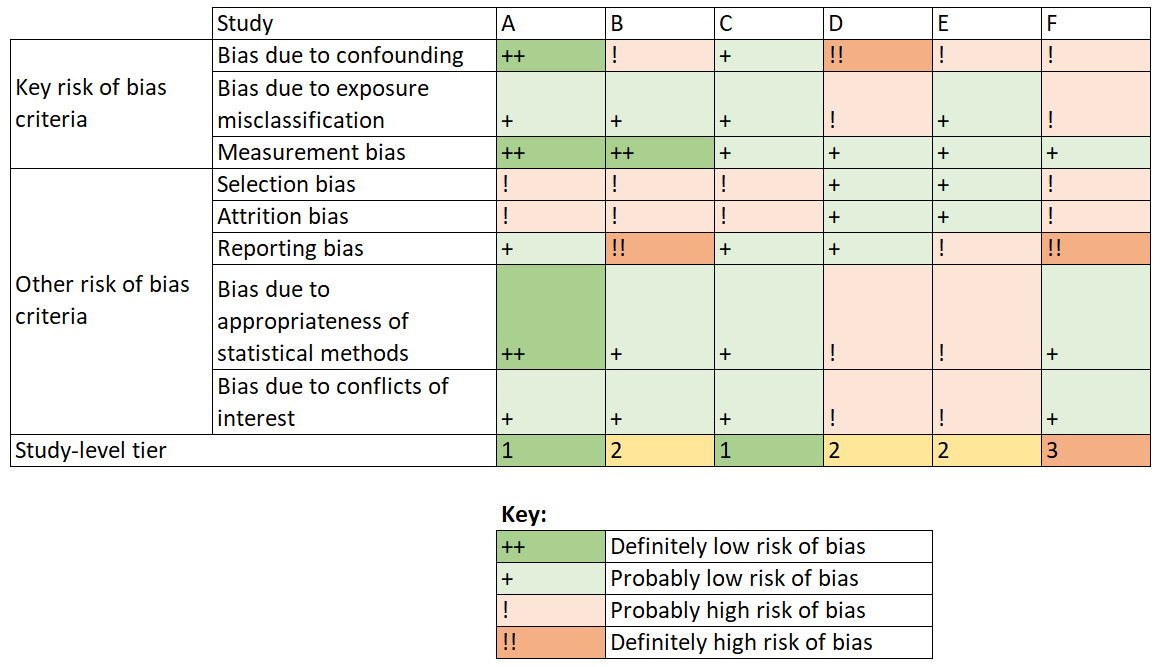


Analysis and synthesis

A theory on how the exposures work will be informed by the DAG (Figure S1) and mechanisms proposed that link exposures with outcomes. A preliminary synthesis will have a textual description of the included studies. Included studies will also be grouped by similarities in their PECO components. For example, studies examining relative humidity and absolute humidity as exposures could be grouped together. Such groupings facilitate patterns between and within the studies to be identified, to allow a thematic analysis of the included studies.

If data from at least four studies with similar or identical outcome and exposure are available, a meta-analysis using random effects models will be conducted. Each meteorological factor and outcome pair will be evaluated separately. Studies will be compared by sample size, place of study, study population, study duration, and exposure assessment, and will be visualised using a forest plot.

To assess inconsistency, an I^2^ value will be calculated for the meta-analyses. This value is the proportion of the variation in effect estimates between studies attributed to real differences between studies, and not due to chance. To aid judgement of inconsistency between studies, an evaluation of forest plots would be necessary. For instance, for two exposure-outcome pairs with identical I^2^ values, the pair with studies differing mainly in effect sizes would be less likely to be downgraded than the pair with a mix of studies suggesting beneficial and harmful effects (Guyatt et al., 2011).

Publication bias will be assessed by conducting Egger’s regression test, and visualisation via funnel plots (Egger et al., 1997). Should fewer than ten studies be used in the meta-analysis, publication bias will not be assessed. These methods used to evaluate publication bias would be underpowered, and funnel plots cannot adequately pick up publication bias among a small number of studies (Lau et al., 2006).

Given sufficient data from the individual studies, sensitivity analyses will be conducted. One of the objectives of sensitivity analyses is to evaluate the magnitude and direction of bias contributed by studies of poorer internal validity. As such, studies assigned a "Tier 3” rating from internal validity assessment would be excluded in the sensitivity analysis, and the resulting effect estimate evaluated. All analyses will be conducted in the most recent version of R.

**Literature**

Alimohammadi, M., & Naderi, M. (2021). Effectiveness of ozone gas on airborne virus inactivation in enclosed spaces: a review study. *Ozone: Science & Engineering*, *43*(1), 21–31.

Aranow, C. (2011). Vitamin D and the immune system. *J Investig Med*, *59*(6), 881–886. <https://doi.org/10.2310/JIM.0b013e31821b8755>

CADTH. (2025). *Grey matters: A tool for searching health-related grey literature*. Retrieved 26.05.2025 from <https://greymatters.cda-amc.ca/>

Charland, K. M., Buckeridge, D. L., Sturtevant, J. L., Melton, F., Reis, B. Y., Mandl, K. D., & Brownstein, J. S. (2009). Effect of environmental factors on the spatio-temporal patterns of influenza spread. *Epidemiol Infect*, *137*(10), 1377–1387. <https://doi.org/https://doi.org/10.1017/s0950268809002283>

Dave, K., & Lee, P. C. (2019). Global Geographical and Temporal Patterns of Seasonal Influenza and Associated Climatic Factors. *Epidemiol Rev*, *41*(1), 51–68. <https://doi.org/10.1093/epirev/mxz008>

Du Ry van Beest Holle, M., Meijer, A., Koopmans, M., & de Jager, C. M. (2005). Human-to-human transmission of avian influenza A/H7N7, The Netherlands, 2003. *Euro Surveill*, *10*(12), 3–4. <https://doi.org/10.2807/esm.10.12.00584-en>

Egger, M., Davey Smith, G., Schneider, M., & Minder, C. (1997). Bias in meta-analysis detected by a simple, graphical test. *Bmj*, *315*(7109), 629–634. <https://doi.org/10.1136/bmj.315.7109.629>

Eick, S. M., Goin, D. E., Chartres, N., Lam, J., & Woodruff, T. J. (2020). Assessing risk of bias in human environmental epidemiology studies using three tools: different conclusions from different tools. *Syst Rev*, *9*(1), 249. <https://doi.org/10.1186/s13643-020-01490-8>

Fagnoul, D., Pasquier, P., Bodson, L., Ortiz, J. A., Vincent, J. L., & De Backer, D. (2013). Myocardial dysfunction during H1N1 influenza infection. *J Crit Care*, *28*(4), 321–327. <https://doi.org/10.1016/j.jcrc.2013.01.010>

Feng, C., Li, J., Sun, W., Zhang, Y., & Wang, Q. (2016). Impact of ambient fine particulate matter (PM2.5) exposure on the risk of influenza-like-illness: a time-series analysis in Beijing, China. *Environ Health*, *15*, 17. <https://doi.org/10.1186/s12940-016-0115-2>

Frampton, G., Whaley, P., Bennett, M., Bilotta, G., Dorne, J.-L. C., Eales, J., James, K., Kohl, C., Land, M., & Livoreil, B. (2022). Principles and framework for assessing the risk of bias for studies included in comparative quantitative environmental systematic reviews. *Environmental evidence*, *11*(1), 12.

Godin, K., Stapleton, J., Kirkpatrick, S. I., Hanning, R. M., & Leatherdale, S. T. (2015). Applying systematic review search methods to the grey literature: a case study examining guidelines for school-based breakfast programs in Canada. *Syst Rev*, *4*, 138. <https://doi.org/10.1186/s13643-015-0125-0>

Guyatt, G. H., Oxman, A. D., Kunz, R., Woodcock, J., Brozek, J., Helfand, M., Alonso-Coello, P., Glasziou, P., Jaeschke, R., Akl, E. A., Norris, S., Vist, G., Dahm, P., Shukla, V. K., Higgins, J., Falck-Ytter, Y., & Schünemann, H. J. (2011). GRADE guidelines: 7. Rating the quality of evidence--inconsistency. *J Clin Epidemiol*, *64*(12), 1294–1302. <https://doi.org/10.1016/j.jclinepi.2011.03.017>

He, Y., Liu, W. J., Jia, N., Richardson, S., & Huang, C. (2023). Viral respiratory infections in a rapidly changing climate: the need to prepare for the next pandemic. *EBioMedicine*, *93*, 104593. <https://doi.org/10.1016/j.ebiom.2023.104593>

Hossain, M. Z., Bambrick, H., Wraith, D., Tong, S., Khan, A. F., Hore, S. K., & Hu, W. (2019). Sociodemographic, climatic variability and lower respiratory tract infections: a systematic literature review. *International journal of biometeorology*, *63*, 209–219.

IPCC. (2023). *Climate Change 2023: Synthesis Report* (Contribution of Working Groups I, II and III to the Sixth Assessment Report of the Intergovernmental Panel on Climate Change (IPCC), Issue.

Kandun, I. N., Wibisono, H., Sedyaningsih, E. R., Yusharmen, Hadisoedarsuno, W., Purba, W., Santoso, H., Septiawati, C., Tresnaningsih, E., Heriyanto, B., Yuwono, D., Harun, S., Soeroso, S., Giriputra, S., Blair, P. J., Jeremijenko, A., Kosasih, H., Putnam, S. D., Samaan, G.,…Uyeki, T. M. (2006). Three Indonesian clusters of H5N1 virus infection in 2005. *N Engl J Med*, *355*(21), 2186–2194. <https://doi.org/10.1056/NEJMoa060930>

Lau, J., Ioannidis, J. P., Terrin, N., Schmid, C. H., & Olkin, I. (2006). The case of the misleading funnel plot. *Bmj*, *333*(7568), 597–600. <https://doi.org/10.1136/bmj.333.7568.597>

Lindner-Cendrowska, K., & Bröde, P. (2021). Impact of biometeorological conditions and air pollution on influenza-like illnesses incidence in Warsaw. *Int J Biometeorol*, *65*(6), 929–944. <https://doi.org/10.1007/s00484-021-02076-2>

NASA. (2025). *Solar Irradiance*. Retrieved 2025/11/22 from <https://earth.gsfc.nasa.gov/climate/projects/solar-irradiance>

Nielsen, J., Mazick, A., Glismann, S., & Mølbak, K. (2011). Excess mortality related to seasonal influenza and extreme temperatures in Denmark, 1994-2010. *BMC Infect Dis*, *11*, 350. <https://doi.org/10.1186/1471-2334-11-350>

NTP. (2015). *OHAT Risk of Bias Rating Tool for Human and Animal Studies*. Retrieved 20.07.2024 from <https://ntp.niehs.nih.gov/go/riskbias>

NTP. (2019). Handbook for conducting a literature-based health assessment using OHAT approach for systematic review and evidence integration. *US Department of Health and Human Services. Available online:* [*https://ntp*](https://ntp)*. niehs. nih. gov/whatwestudy/assessments/noncancer/handbook/index. html*.

Paez, A. (2017). Gray literature: An important resource in systematic reviews. *J Evid Based Med*, *10*(3), 233–240. <https://doi.org/10.1111/jebm.12266>

Pappas, C., & Williams, I. (2011). Grey literature: its emerging importance. *Journal of Hospital Librarianship*, *11*(3), 228–234.

Park, J. E., Son, W. S., Ryu, Y., Choi, S. B., Kwon, O., & Ahn, I. (2020). Effects of temperature, humidity, and diurnal temperature range on influenza incidence in a temperate region. *Influenza Other Respir Viruses*, *14*(1), 11–18. <https://doi.org/10.1111/irv.12682>

Peci, A., Winter, A. L., Li, Y., Gnaneshan, S., Liu, J., Mubareka, S., & Gubbay, J. B. (2019). Effects of Absolute Humidity, Relative Humidity, Temperature, and Wind Speed on Influenza Activity in Toronto, Ontario, Canada. *Appl Environ Microbiol*, *85*(6). <https://doi.org/10.1128/aem.02426-18>

Rooney, A. A., Cooper, G. S., Jahnke, G. D., Lam, J., Morgan, R. L., Boyles, A. L., Ratcliffe, J. M., Kraft, A. D., Schünemann, H. J., Schwingl, P., Walker, T. D., Thayer, K. A., & Lunn, R. M. (2016). How credible are the study results? Evaluating and applying internal validity tools to literature-based assessments of environmental health hazards. *Environ Int*, *92-93*, 617–629. <https://doi.org/10.1016/j.envint.2016.01.005>

Roussel, M., Pontier, D., Cohen, J. M., Lina, B., & Fouchet, D. (2016). Quantifying the role of weather on seasonal influenza. *BMC Public Health*, *16*, 441. <https://doi.org/https://doi.org/10.1186/s12889-016-3114-x>

Sagripanti, J. L., & Lytle, C. D. (2007). Inactivation of influenza virus by solar radiation. *Photochem Photobiol*, *83*(5), 1278–1282. <https://doi.org/https://doi.org/10.1111/j.1751-1097.2007.00177.x>

Spencer, J. A., Shutt, D. P., Moser, S. K., Clegg, H., Wearing, H. J., Mukundan, H., & Manore, C. A. (2022). Distinguishing viruses responsible for influenza-like illness. *J Theor Biol*, *545*, 111145. <https://doi.org/10.1016/j.jtbi.2022.111145>

Steenland, K., Schubauer-Berigan, M. K., Vermeulen, R., Lunn, R. M., Straif, K., Zahm, S., Stewart, P., Arroyave, W. D., Mehta, S. S., & Pearce, N. (2020). Risk of Bias Assessments and Evidence Syntheses for Observational Epidemiologic Studies of Environmental and Occupational Exposures: Strengths and Limitations. *Environ Health Perspect*, *128*(9), 95002. <https://doi.org/10.1289/ehp6980>

Stellrecht, K. (2017). Molecular testing for respiratory viruses. In *Diagnostic molecular pathology* (pp. 123–137). Elsevier.

Suntronwong, N., Vichaiwattana, P., Klinfueng, S., Korkong, S., Thongmee, T., Vongpunsawad, S., & Poovorawan, Y. (2020). Climate factors influence seasonal influenza activity in Bangkok, Thailand. *PLoS One*, *15*(9), e0239729. <https://doi.org/10.1371/journal.pone.0239729>

Tamerius, J. D., Shaman, J., Alonso, W. J., Bloom-Feshbach, K., Uejio, C. K., Comrie, A., & Viboud, C. (2013). Environmental predictors of seasonal influenza epidemics across temperate and tropical climates. *PLoS Pathog*, *9*(3), e1003194. <https://doi.org/https://doi.org/10.1371/journal.ppat.1003194>

Toczylowski, K., Wietlicka-Piszcz, M., Grabowska, M., & Sulik, A. (2021). Cumulative Effects of Particulate Matter Pollution and Meteorological Variables on the Risk of Influenza-Like Illness. *Viruses*, *13*(4). <https://doi.org/https://doi.org/10.3390/v13040556>

Tseng, C., & Li, C. (2008). Inactivation of surface viruses by gaseous ozone. *J Environ Health*, *70*(10), 56–62.

Viswanathan, M., Ansari, M., Berkman, N., Chang, S., Hartling, L., McPheeters, M., & Treadwell, J. (2012). Assessing the risk of bias of individual studies in systematic reviews of health care interventions. Agency for healthcare research and quality methods guide for comparative effectiveness reviews. *AHRQ Methods for Effective Health Care*.

Walker, T. A., Waite, B., Thompson, M. G., McArthur, C., Wong, C., Baker, M. G., Wood, T., Haubrock, J., Roberts, S., Gross, D. K., Huang, Q. S., & Newbern, E. C. (2020). Risk of Severe Influenza Among Adults With Chronic Medical Conditions. *J Infect Dis*, *221*(2), 183–190. <https://doi.org/10.1093/infdis/jiz570>

Whaley, P., Aiassa, E., Beausoleil, C., Beronius, A., Bilotta, G., Boobis, A., de Vries, R., Hanberg, A., Hoffmann, S., & Hunt, N. (2020). Recommendations for the conduct of systematic reviews in toxicology and environmental health research (COSTER). *Environment international*, *143*, 105926.

Yan, Z. L., Liu, W. H., Long, Y. X., Ming, B. W., Yang, Z., Qin, P. Z., Ou, C. Q., & Li, L. (2024). Effects of meteorological factors on influenza transmissibility by virus type/subtype. *BMC Public Health*, *24*(1), 494. <https://doi.org/10.1186/s12889-024-17961-9>

Zhang, B., Chen, T., Liang, S., Shen, W., Sun, Q., Wang, D., Wang, G., Yang, J., Yang, L., Wang, D., Shu, Y., & Du, X. (2022). Subtypes specified environmental dependence of seasonal influenza virus. *Sci Total Environ*, *852*, 158525. <https://doi.org/https://doi.org/10.1016/j.scitotenv.2022.158525>

Zhong, Y., Qin, Y., Yu, H., Yu, J., Wu, H., Chen, L., Zhang, P., Wang, X., Jia, Z., Guo, Y., Zhang, H., Shan, J., Wang, Y., Xie, H., Li, X., & Li, Z. (2015). Avian influenza virus infection risk in humans with chronic diseases. *Sci Rep*, *5*, 8971. <https://doi.org/10.1038/srep08971>

**Supplementary Materials S2 – Search Strategy**

**PubMed**: 3,347 hits

((“Influenza, Human"[Mesh]) OR ("Influenzavirus A"[Mesh]) OR ("Influenzavirus B"[Mesh]) OR ("Influenzavirus C"[Mesh]) OR ("Influenza-like"[Title/Abstract]) OR (“influenza like” [Title/Abstract]) OR ("Influenza virus" [Title/Abstract]) OR (“influenzavirus” [Title/Abstract]) OR (“influenza-virus” [Title/Abstract]) OR ("influenza B" [Title/Abstract]) OR (“influenza virus B” [Title/Abstract]) OR (“influenzavirus B” [Title/Abstract]) OR (“influenza-virus B” [Title/Abstract]) OR ("influenza A" [Title/Abstract]) OR (“influenza virus A” [Title/Abstract]) OR (“influenzavirus A” [Title/Abstract]) OR (“influenza-virus A” [Title/Abstract]) OR (“influenza C” [Title/Abstract]) OR (“influenza virus C” [Title/Abstract]) OR (“influenzavirus C” [Title/Abstract]) OR (“influenza-virus C” [Title/Abstract]) OR (“flu” [Title/Abstract]))

AND (("Temperature"[Mesh]) OR ("Humidity"[Mesh]) OR ("Rain"[Mesh]) OR ("Cold Temperature"[Mesh]) OR ("Hot temperature"[Mesh]) OR ("Extreme Heat"[Mesh]) OR (“Wind”[Mesh]) OR (“Ultraviolet Rays”[Mesh]) OR ("hot spell*" [Title/Abstract]) OR ("hot-spell*" [Title/Abstract]) OR ("hotspell*" [Title/Abstract]) OR ("heat" [Title/Abstract]) OR (“heat related” [Title/Abstract]) OR ("heat-related" [Title/Abstract]) OR ("cold spell*" [Title/Abstract]) OR ("cold-spell*" [Title/Abstract]) OR ("coldspell*" [Title/Abstract]) OR ("cold" [Title/Abstract]) OR ("cold-related" [Title/Abstract]) OR ("cold related" [Title/Abstract]) OR ("cold temperature*" [Title/Abstract]) OR ("temperature*" [Title/Abstract]) OR ("ambient temperature*" [Title/Abstract]) OR ("outdoor temperature*" [Title/Abstract]) OR ("air temperature*" [Title/Abstract]) OR ("apparent temperature*" [Title/Abstract]) OR ("diurnal temperature*" [Title/Abstract]) OR ("temperature-related" [Title/Abstract]) OR ("temperature variability" [Title/Abstract]) OR ("temperature effect*" [Title/Abstract]) OR ("hot temperature*" [Title/Abstract]) OR ("high temperature*" [Title/Abstract]) OR ("temperature extreme*" [Title/Abstract]) OR ("Extreme temperature*" [Title/Abstract]) OR ("low temperature*" [Title/Abstract]) OR ("surface temperature*" [Title/Abstract]) OR ("surface-temperature*" [Title/Abstract]) OR ("ground level temperature*" [Title/Abstract]) OR ("ground-level temperature*" [Title/Abstract]) OR ("weather" [Title/Abstract]) OR (“climat*” [Title/Abstract]) OR ("humidity" [Title/Abstract]) OR (“rain” [Title/Abstract]) OR ("rainfall" [Title/Abstract]) OR ("precipitation" [Title/Abstract]) OR ("wind" [Title/Abstract]) OR ("wind velocity" [Title/Abstract]) OR ("wind-velocity" [Title/Abstract]) OR ("windspeed" [Title/Abstract]) OR ("wind-speed" [Title/Abstract]) OR ("wind speed" [Title/Abstract]) OR ("absolute humidity" [Title/Abstract]) OR ("relative humidity" [Title/Abstract]) OR ("thermal stress" [Title/Abstract]) OR ("thermal-stress" [Title/Abstract]) OR ("heat stress" [Title/Abstract]) OR ("heat-stress" [Title/Abstract]) OR ("cold stress" [Title/Abstract]) OR ("cold-stress" [Title/Abstract]) OR ("UV light" [Title/Abstract]) OR ("UV-light" [Title/Abstract]) OR ("UVlight" [Title/Abstract]) OR ("UV radiation" [Title/Abstract]) OR ("UV-radiation" [Title/Abstract]) OR ("ultraviolet*" [Title/Abstract]) OR ("ultra violet*" [Title/Abstract]) OR ("ultra-violet*" [Title/Abstract]) OR ("ultraviolet ray*" [Title/Abstract]) OR ("ultraviolet-ray*" [Title/Abstract]) OR ("solar radiation" [Title/Abstract]) OR ("solar-radiation" [Title/Abstract]) OR ("UV-index" [Title/Abstract]) OR ("UV Index" [Title/Abstract]))

NOT ((“virus like") OR ("virus-like") OR ("culture") OR ("cell-lines") OR ("cell-line") OR ("in-vitro") OR ("in vitro") or ("heat shock") OR ("heat-shock") OR ("heat shocked") OR ("heat-shocked") OR ("vaccine") OR ("Influenza Vaccines"[Mesh]) OR ("Influenza, Human/drug therapy"[Mesh]) OR (“body temperature” [Mesh]))

**Web of Science**: 4,280 hits

#1 (TI=((("Human Influenza*") OR ("Human-Influenza*") OR ("influenza*") OR (“influenza-virus”) OR ("influenza B") OR (“influenza virus B”) OR (“influenzavirus B”) OR (“influenza-virus B”) OR ("influenza A") OR (“influenza virus A”) OR (“influenzavirus A”) OR (“influenza-virus A”) OR (“influenza C”) OR (“influenza virus C”) OR (“influenzavirus C”) OR (“influenza-virus C”) OR (“flu”) OR ("influenza-like") OR ("influenzalike") OR ("influenza like")))

OR AB=((("Human Influenza*") OR ("Human-Influenza*") OR ("influenza*") OR (“influenza-virus”) OR ("influenza B") OR (“influenza virus B”) OR (“influenzavirus B”) OR (“influenza-virus B”) OR ("influenza A") OR (“influenza virus A”) OR (“influenzavirus A”) OR (“influenza-virus A”) OR (“influenza C”) OR (“influenza virus C”) OR (“influenzavirus C”) OR (“influenza-virus C”) OR (“flu”) OR ("influenza-like") OR ("influenzalike") OR ("influenza like"))))

**#2 (TI=((("Humidity") OR ("Rain") OR (“climat*”) OR (“Extreme Heat”) OR (“hot spell*”) OR (“hot-spell*”) OR (“hotspell*”) OR ("heat") OR (“heat related”) OR (“heat-related”) OR (“cold spell*”) OR (“cold-spell*”) OR (“coldspell*”) OR (“cold”) OR (“cold-related”) OR (“cold related”) OR (“cold temperature*”) OR (“temperature*") OR (“ambient temperature*”) OR (“outdoor temperature*”) OR (“air temperature*”) OR (“apparent temperature*”) OR (“diurnal temperature*”) OR (“temperature-related”) OR (“temperature variability”) OR (“temperature effect*”))) OR**

**AB=((("Humidity") OR ("Rain") OR (“climat*”) OR (“Extreme Heat”) OR (“hot spell*”) OR (“hot-spell*”) OR (“hotspell*”) OR ("heat") OR (“heat related”) OR (“heat-related”) OR (“cold spell*”) OR (“cold-spell*”) OR (“coldspell*”) OR (“cold”) OR (“cold-related”) OR (“cold related”) OR (“cold temperature*”) OR (“temperature*") OR (“ambient temperature*”) OR (“outdoor temperature*”) OR (“air temperature*”) OR (“apparent temperature*”) OR (“diurnal temperature*”) OR (“temperature-related”) OR (“temperature variability”) OR (“temperature effect*”))))**

**#3 (TI=(((“hot temperature*”) OR (“high temperature*”) OR (“temperature extreme*”) OR (“Extreme temperature*”) OR (“low temperature*”) OR (“surface temperature*”) OR (“surface-temperature*”) OR (“ground level temperature*”) OR (“ground-level temperature*”) OR ("weather") OR ("rainfall") OR ("precipitation") OR ("wind") OR (“wind velocity”) OR (“wind-velocity”) OR (“windspeed”) OR (“wind-speed”) OR (“wind speed”) OR ("air pressure") OR (“absolute humidity”) OR (“relative humidity”) OR (“thermal stress”) OR (“thermal-stress”) OR (“heat stress”) OR (“heat-stress”) OR (“cold stress”) OR (“cold-stress”) OR (“UV light”) OR (“UV-light”) OR (“UVlight”) OR (“UV radiation”) OR (“UV-radiation”) OR (“ultraviolet*”) OR (“ultra violet*”) OR (“ultra-violet*”) OR (“ultraviolet ray”) OR (“ultraviolet-ray*”) OR (“solar radiation”) OR (“solar-radiation”) OR (“UV-index”) OR (“UV Index”))) OR**

**AB=(((“hot temperature*”) OR (“high temperature*”) OR (“temperature extreme*”) OR (“Extreme temperature*”) OR (“low temperature*”) OR (“surface temperature*”) OR (“surface-temperature*”) OR (“ground level temperature*”) OR (“ground-level temperature*”) OR ("weather") OR ("rainfall") OR ("precipitation") OR ("wind") OR (“wind velocity”) OR (“wind-velocity”) OR (“windspeed”) OR (“wind-speed”) OR (“wind speed”) OR ("air pressure") OR (“absolute humidity”) OR (“relative humidity”) OR (“thermal stress”) OR (“thermal-stress”) OR (“heat stress”) OR (“heat-stress”) OR (“cold stress”) OR (“cold-stress”) OR (“UV light”) OR (“UV-light”) OR (“UVlight”) OR (“UV radiation”) OR (“UV-radiation”) OR (“ultraviolet*”) OR (“ultra violet*”) OR (“ultra-violet*”) OR (“ultraviolet ray”) OR (“ultraviolet-ray*”) OR (“solar radiation”) OR (“solar-radiation”) OR (“UV-index”) OR (“UV Index”))))**

#4 TS=(((“virus like") OR ("virus-like") OR ("culture") OR ("cell-line*") OR ("in-vitro") OR ("in vitro") or ("heat shock") OR ("heat-shock") OR ("heat shocked") OR ("heat-shocked") OR ("vaccine*") OR ("Influenza Vaccine*") OR (“influenza-vaccine*”) OR (“influenzavaccine*”) OR ("drug therapy") OR (“drug-therapy”) OR (“drugtherapy”) OR (“body temperature*” ) OR (“body-temperature*”) OR (“bodytemperature*”)))

#5

#1 AND ((#2) OR (#3)) NOT #4

**Scopus**: 6,358 hits

(TITLE-ABS(("Human Influenza*") OR ("Human-Influenza*") OR ("influenza*") OR ("influenza-virus") OR ("influenza B")OR ("influenza virus B") OR ("influenzavirus B") OR ("influenza-virus B") OR ("influenza A") OR ("influenza virus A") OR ("influenzavirus A") OR ("influenza-virus A") OR ("influenza C") OR ("influenza virus C") OR ("influenzavirus C") OR ("influenza-virus C") OR ("flu") OR ("influenza-like") OR ("influenzalike") OR ("influenza like")))

AND (TITLE-ABS(("Humidity") OR ("Rain") OR ("climat*") OR ("Hot temperature*") OR ("Extreme Heat") OR ("hot spell*") OR ("hot-spell*") OR ("hotspell*") OR ("heat") OR ("heat-related") OR ("heat related") OR ("cold spell*") OR ("cold-spell*") OR ("coldspell*") OR ("cold") OR ("cold-related") OR ("cold related") OR ("cold temperature*") OR ("temperature*") OR ("ambient temperature*") OR ("outdoor temperature*") OR ("air temperature*") OR ("apparent temperature*") OR ("diurnal temperature*") OR ("temperature-related") OR ("temperature variability") OR ("temperature effect*") OR ("high temperature*") OR ("temperature extreme*") OR ("Extreme temperature*") OR ("low temperature*") OR ("surface temperature*") OR ("surface-temperature*") OR ("ground level temperature*") OR ("ground-level temperature*") OR ("weather") OR ("rainfall") OR ("precipitation") OR ("wind") OR ("wind velocity") OR ("wind-velocity") OR ("windspeed") OR ("wind-speed") OR ("wind speed") OR ("air pressure") OR ("absolute humidity") OR ("relative humidity") OR ("thermal stress") OR ("thermal-stress") OR ("heat stress") OR ("heat-stress") OR ("cold stress") OR ("cold-stress") OR ("UV light") OR ("UV-light") OR ("UVlight") OR ("UV radiation") OR ("UV-radiation") OR ("ultraviolet*") OR ("ultra violet*") OR ("ultra-violet*") OR ("ultraviolet ray") OR ("ultraviolet-ray*") OR ("solar radiation") OR ("solar-radiation") OR ("UV-index") OR ("UV Index")))

AND NOT (KEY(("virus like") OR ("virus-like") OR ("culture") OR ("cell-line*") OR ("in-vitro") OR ("in vitro") OR ("heat shock") OR ("heat-shock") OR ("heat shocked") OR ("heat-shocked") OR ("vaccine*") OR ("Influenza Vaccine*") OR ("influenza-vaccine*") OR ("influenzavaccine*") OR ("drug therapy") OR ("drug-therapy") OR ("drugtherapy") OR ("body temperature*") OR ("body-temperature*") OR ("bodytemperature*")))

**MedRxiv**: 266 hits

Influenza AND Humidity, Rain, climat*, “hot temperature*”, “extreme heat”, “hot spell*”, heat, “heat related”, “cold spell*”, “cold related”, “cold temperature*”, “temperature*”, “ambient temperature*”, “outdoor temperature*”, “air temperature*”, “apparent temperature*”, “diurnal temperature*”, temperature-related, “temperature variability”, “temperature effect*”, “high temperature*”, “temperature extreme*”, “extreme temperature*”, “low temperature*”, “surface temperature*”, “ground level temperature*”, “weather”, “rainfall”, “precipitation”, “wind”, “wind velocity”, “wind speed”, “air pressure”, “absolute humidity”, “relative humidity”, “thermal stress”, “heat stress”, “cold stress”, “UV light”, “UV radiation”, “ultraviolet”, “ultraviolet ray*”, “solar radiation”, “UV index”

**Supplementary Materials S3 – Spread Sheet Examples**


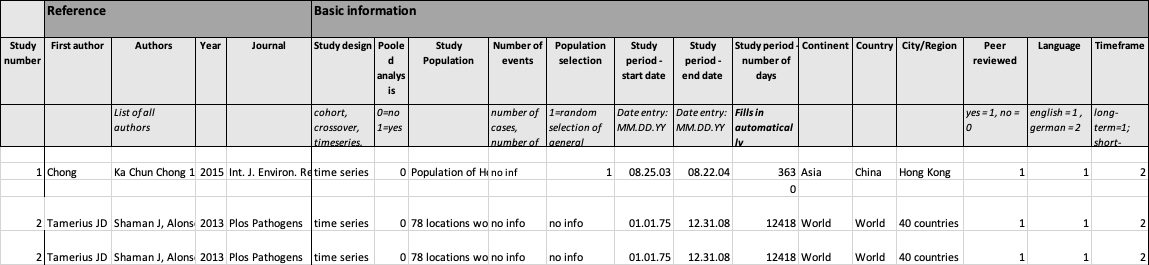


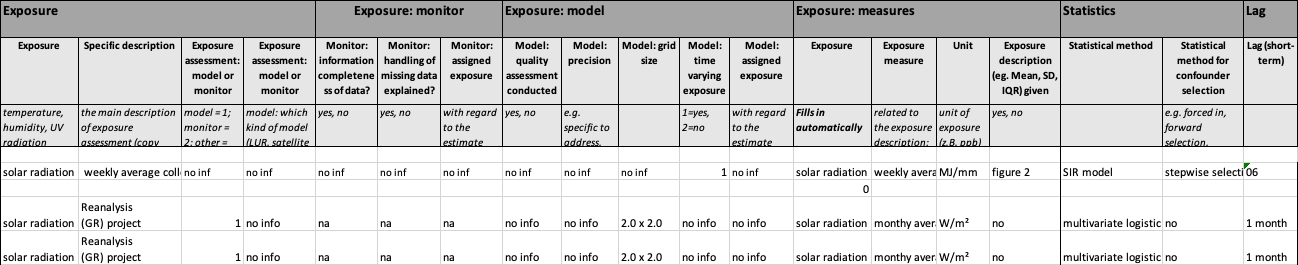


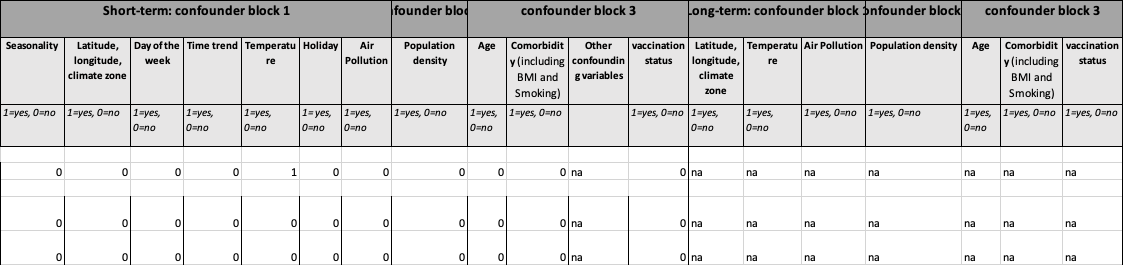


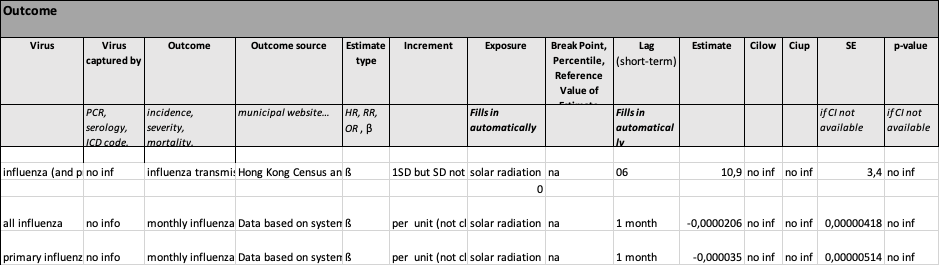


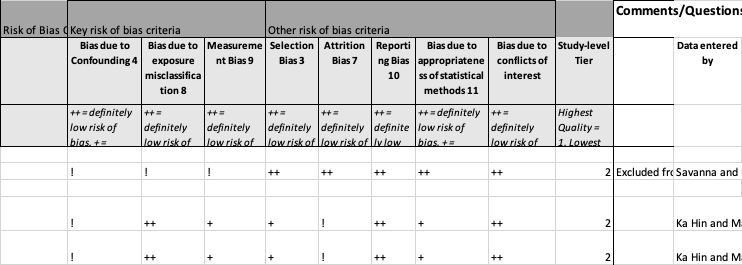


**Supplementary Materials S4 – Sensitivity Analyses**

**Supplementary Figure S3**. Findings from sensitivity analysis for a random-effects meta-analysis using DerSimonian-Laird estimators showing RR and 95% CIs for influenza incidence, corresponding to a change per 1 W/m^2^ increase in solar radiation.


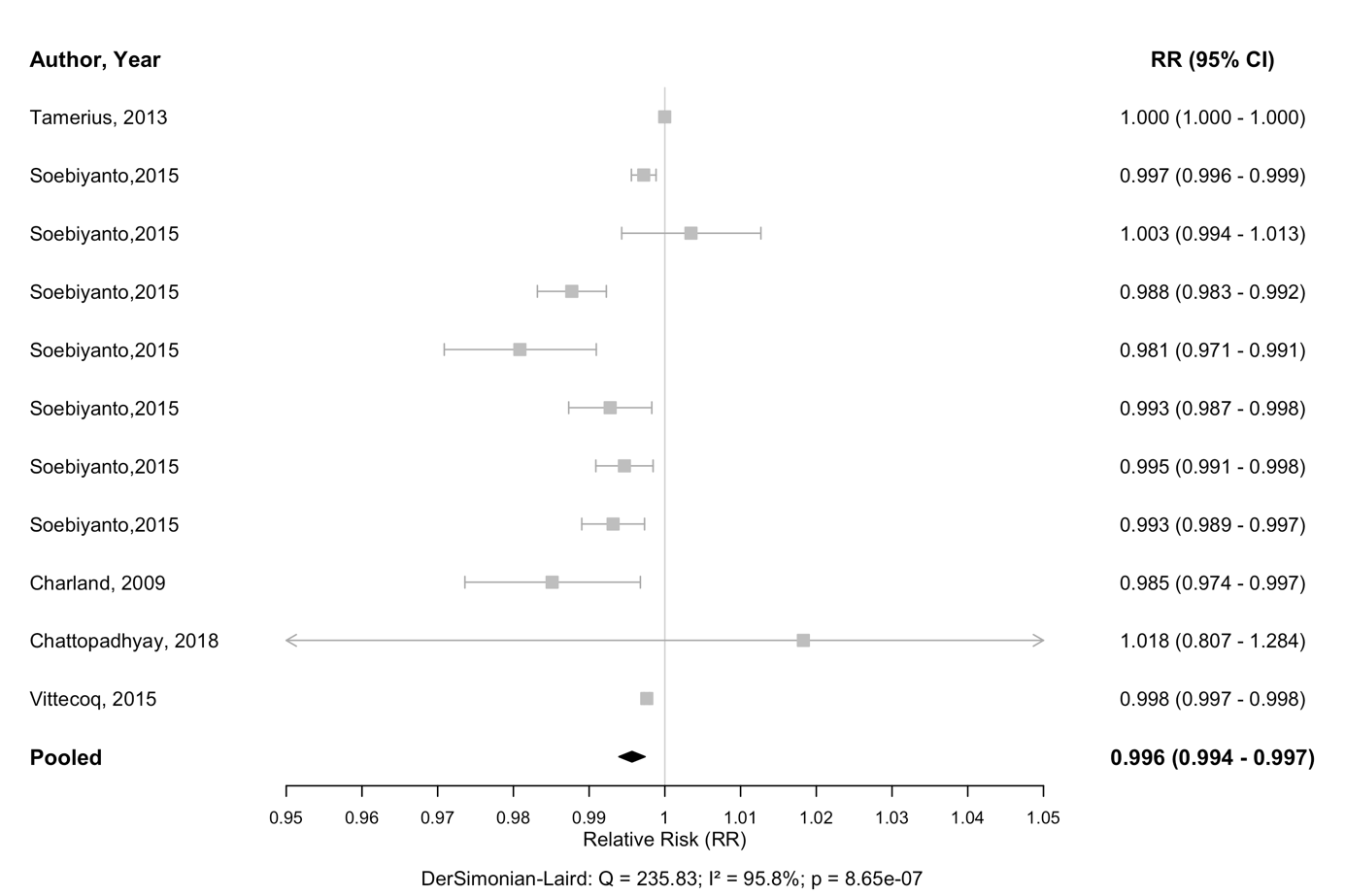


**Figure S3**: Results were presented as relative risks (RR) and 95% confidence intervals (CIs). RE: Random Effects. Q: Cochran’s Q-Statistics. pr_low: lower bound of the 95% prediction interval. pr_up: upper bound of the 95% prediction interval.

**Supplementary Figure S4**. Findings from sensitivity analysis for a random-effects meta-analysis using Paule-Mandel estimators showing RR and 95% CIs for influenza incidence, corresponding to a change per 1 W/m^2^ increase in solar radiation.


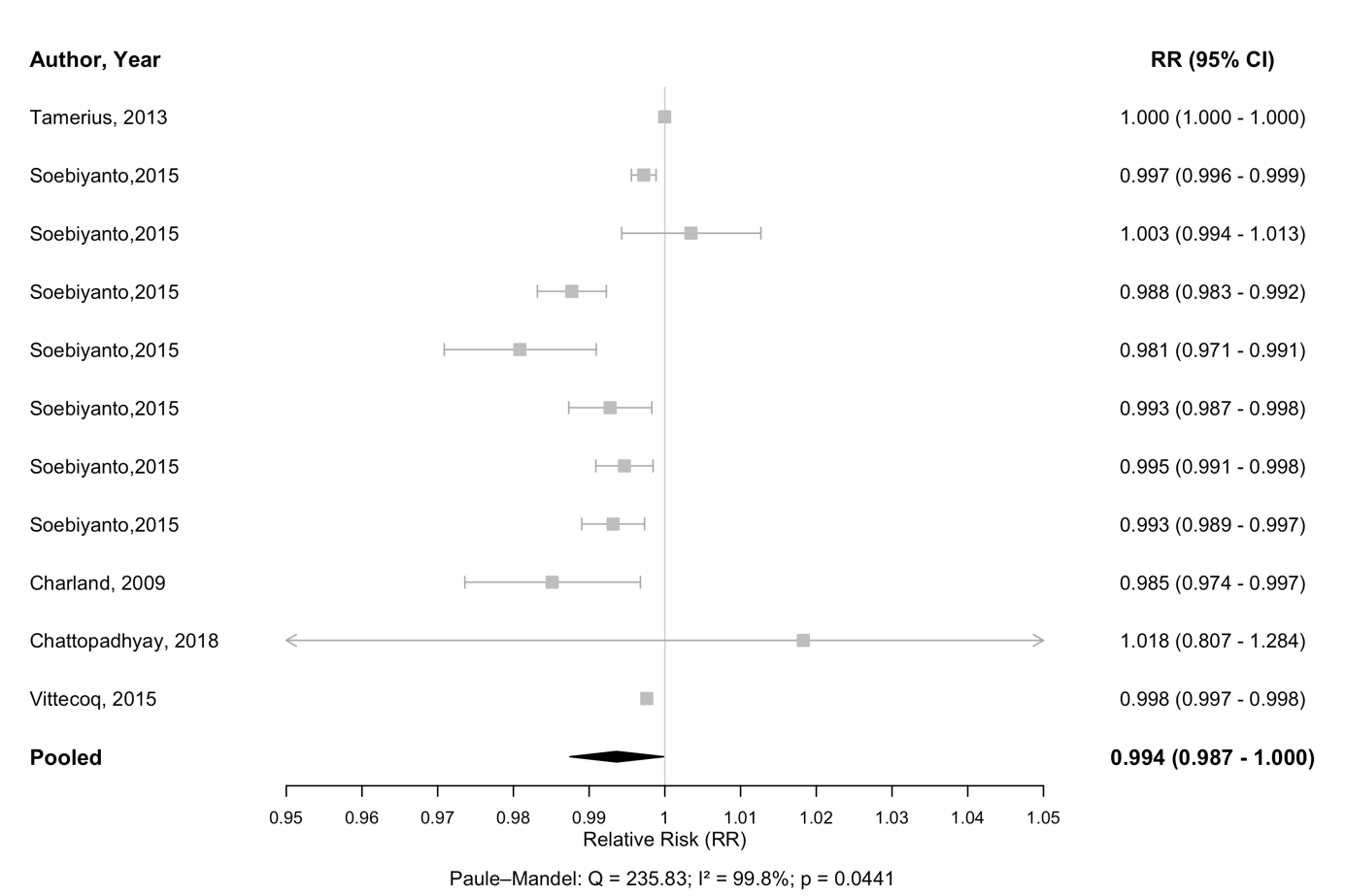


**Figure S4**: Results were presented as relative risks (RR) and 95% confidence intervals (CIs). RE: Random Effects. Q: Cochran’s Q-Statistics. pr_low: lower bound of the 95% prediction interval. pr_up: upper bound of the 95% prediction interval.

**Supplementary Figure S5**. Findings from sensitivity analysis for a random-effects meta-analysis excluding studies which do not provide an increment, showing RR and 95% CIs for influenza incidence, corresponding to a change per 1 W/m^2^ increase in solar radiation.


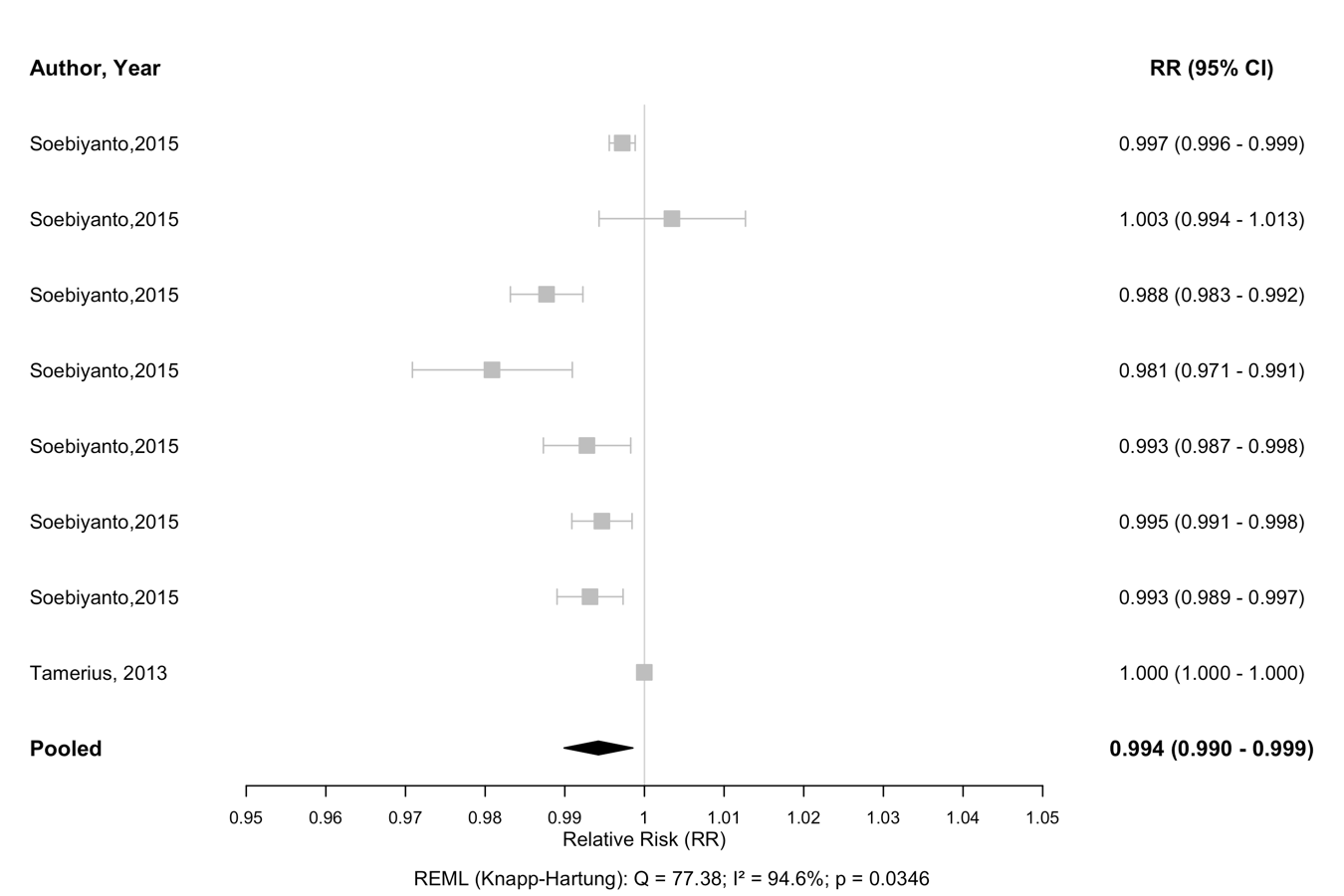


**Figure S5**: Results were presented as relative risks (RR) and 95% confidence intervals (CIs). RE: Random Effects. Q: Cochran’s Q-Statistics. pr_low: lower bound of the 95% prediction interval. pr_up: upper bound of the 95% prediction interval.

**Supplementary Figure S6**. Findings from sensitivity analysis for a random-effects meta-analysis using DerSimonian-Laird estimators showing RR and 95% CIs for influenza incidence, corresponding to a change per 1 hour increase in sunshine duration.


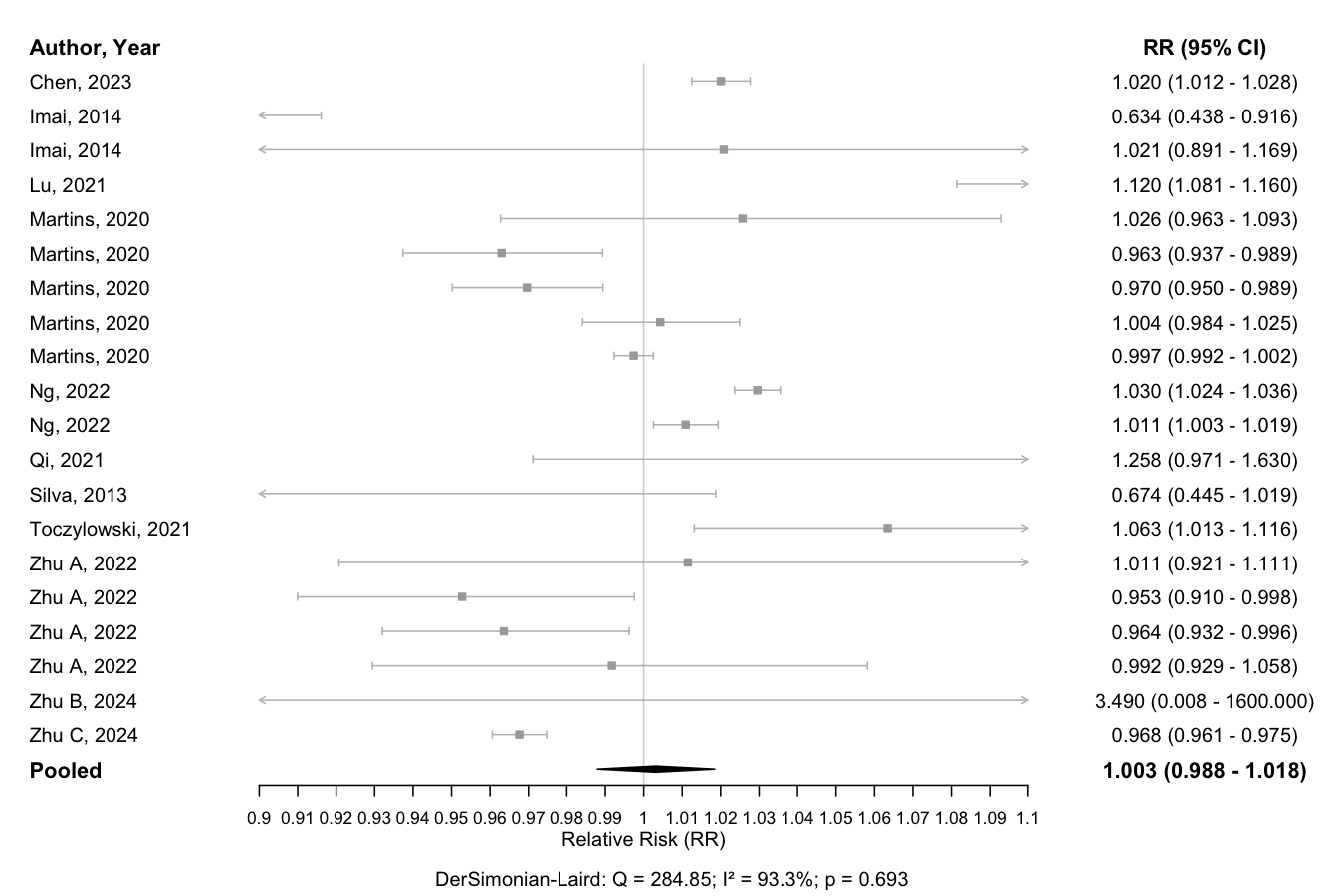


**Figure S6**: Results were presented as relative risks (RR) and 95% confidence intervals (CIs). RE: Random Effects. Q: Cochran’s Q-Statistics. pr_low: lower bound of the 95% prediction interval. pr_up: upper bound of the 95% prediction interval.

**Supplementary Figure S7**. Findings from sensitivity analysis for a random-effects meta-analysis using Paule-Mandel estimators showing RR and 95% CIs for influenza incidence, corresponding to a change per 1 hour increase in sunshine duration.


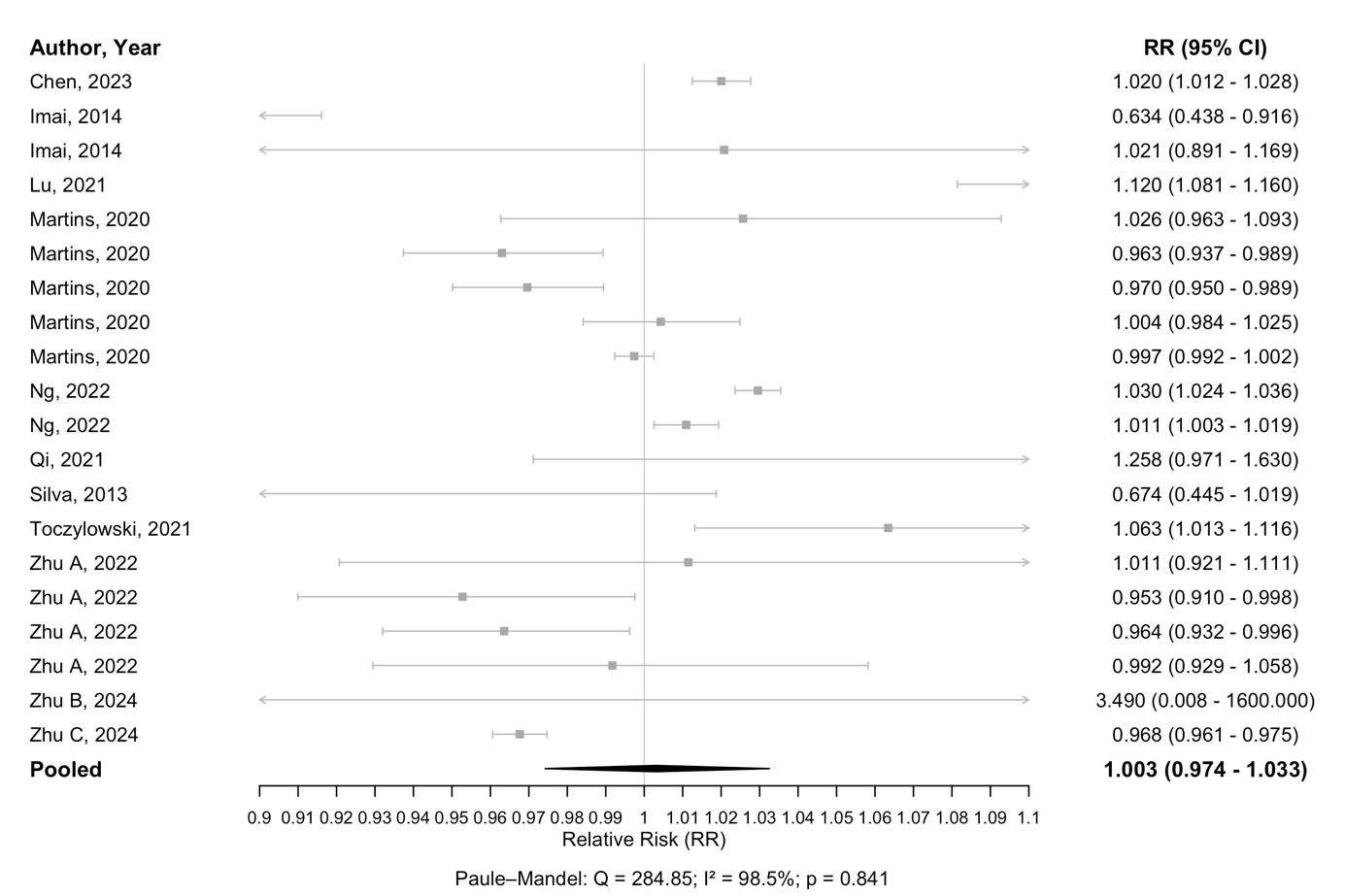


**Figure S7**: Results were presented as relative risks (RR) and 95% confidence intervals (CIs). RE: Random Effects. Q: Cochran’s Q-Statistics. pr_low: lower bound of the 95% prediction interval. pr_up: upper bound of the 95% prediction interval.

**Supplementary Figure S8**. Findings from sensitivity analysis for a random-effects meta-analysis excluding studies which do not provide an increment, showing RR and 95% CIs for influenza incidence, corresponding to a change per 1 hour increase in sunshine duration.


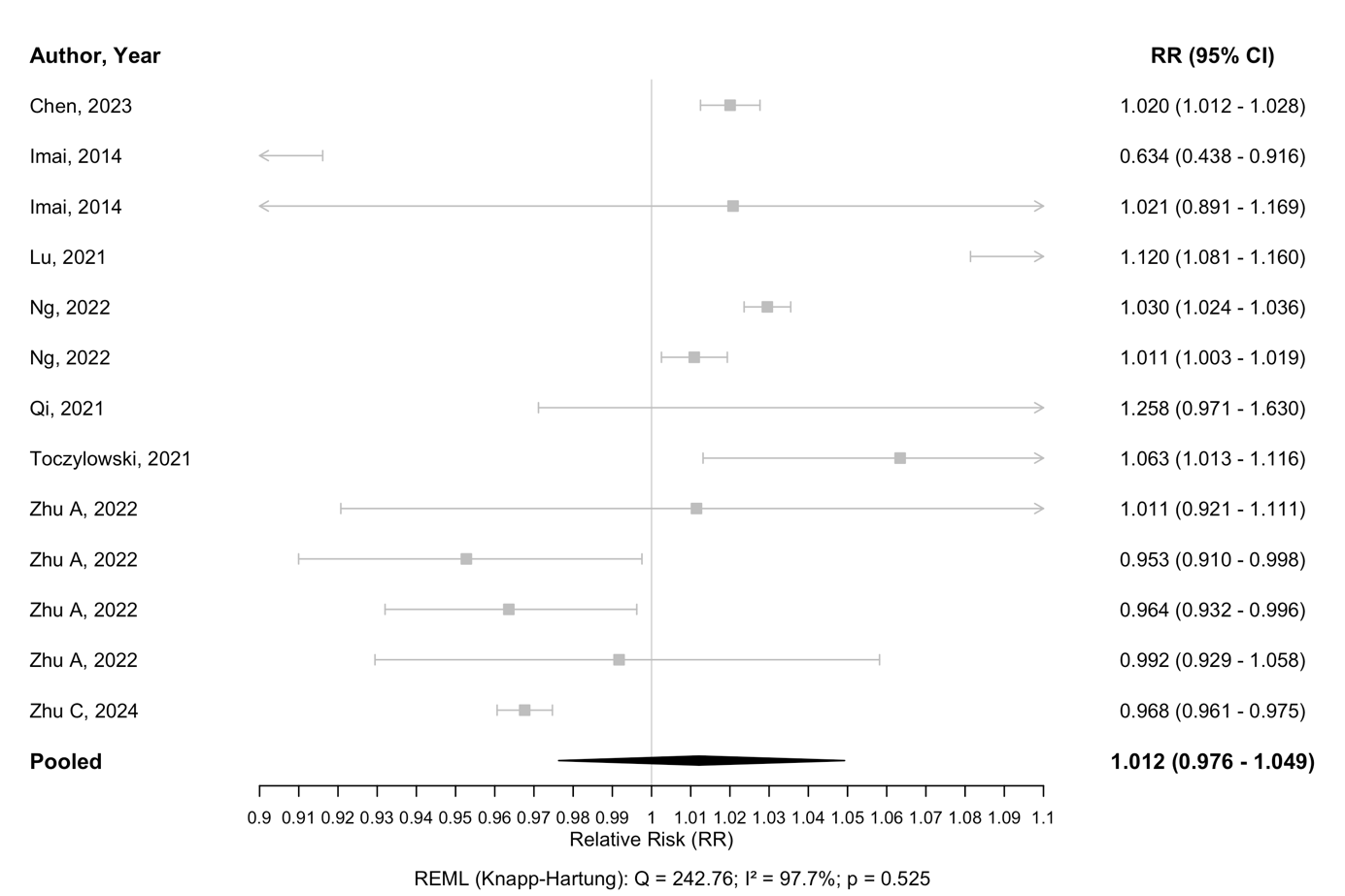


**Figure S8**: Results were presented as relative risks (RR) and 95% confidence intervals (CIs). RE: Random Effects. Q: Cochran’s Q-Statistics. pr_low: lower bound of the 95% prediction interval. pr_up: upper bound of the 95% prediction interval.

**Supplementary Figure S9**. Findings from sensitivity analysis for a random-effects meta-analysis excluding studies with a high RoB, showing RR and 95% CIs for influenza incidence, corresponding to a change per 1 hour increase in sunshine duration.


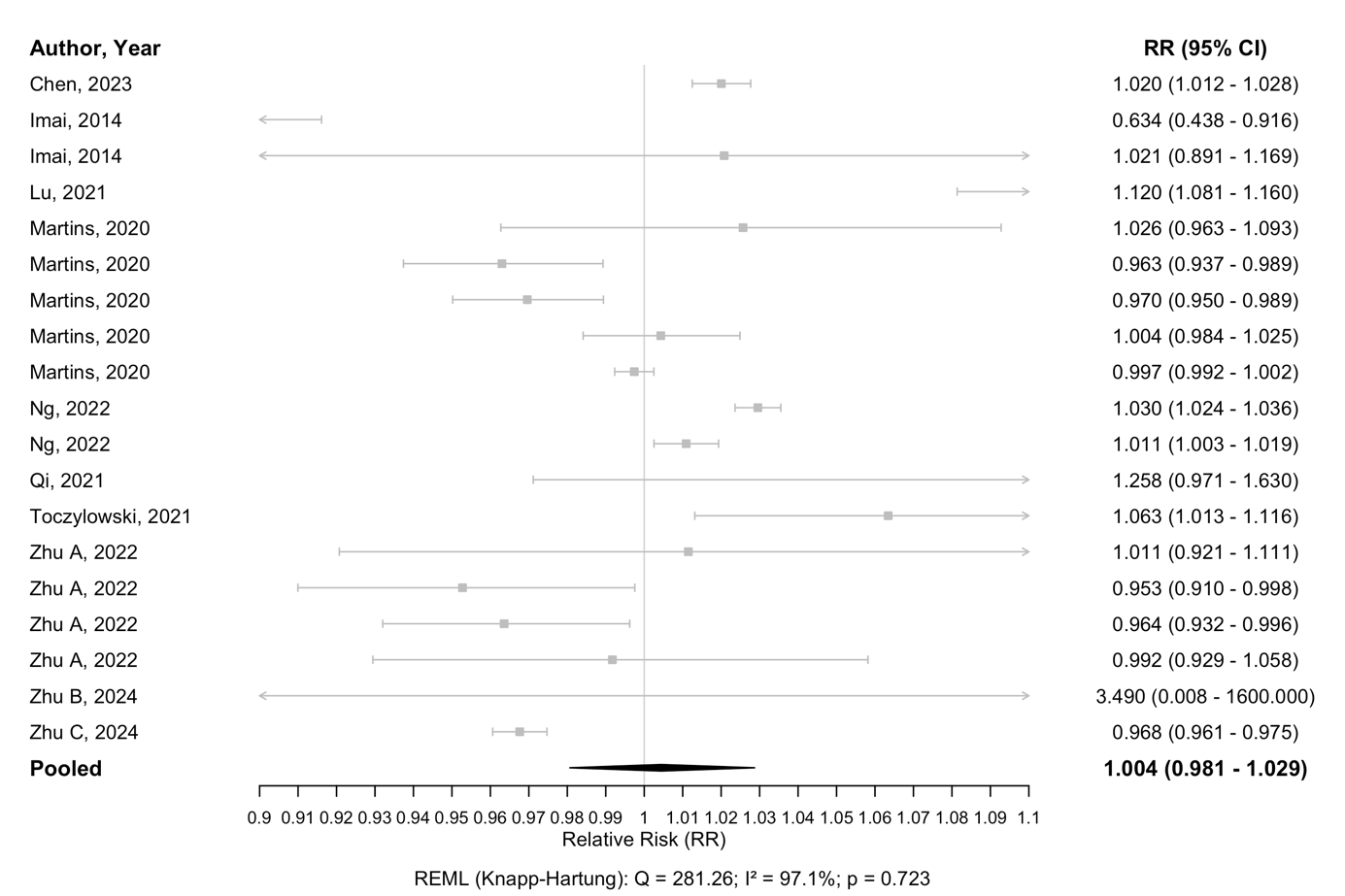


**Figure S9**: Results were presented as relative risks (RR) and 95% confidence intervals (CIs). RE: Random Effects. Q: Cochran’s Q-Statistics. pr_low: lower bound of the 95% prediction interval. pr_up: upper bound of the 95% prediction interval.

**Supplementary Figure S10**. Findings from sensitivity analysis for a random-effects meta-analysis excluding studies using ILI as outcome, showing RR and 95% CIs for influenza incidence, corresponding to a change per 1 hour increase in sunshine duration.


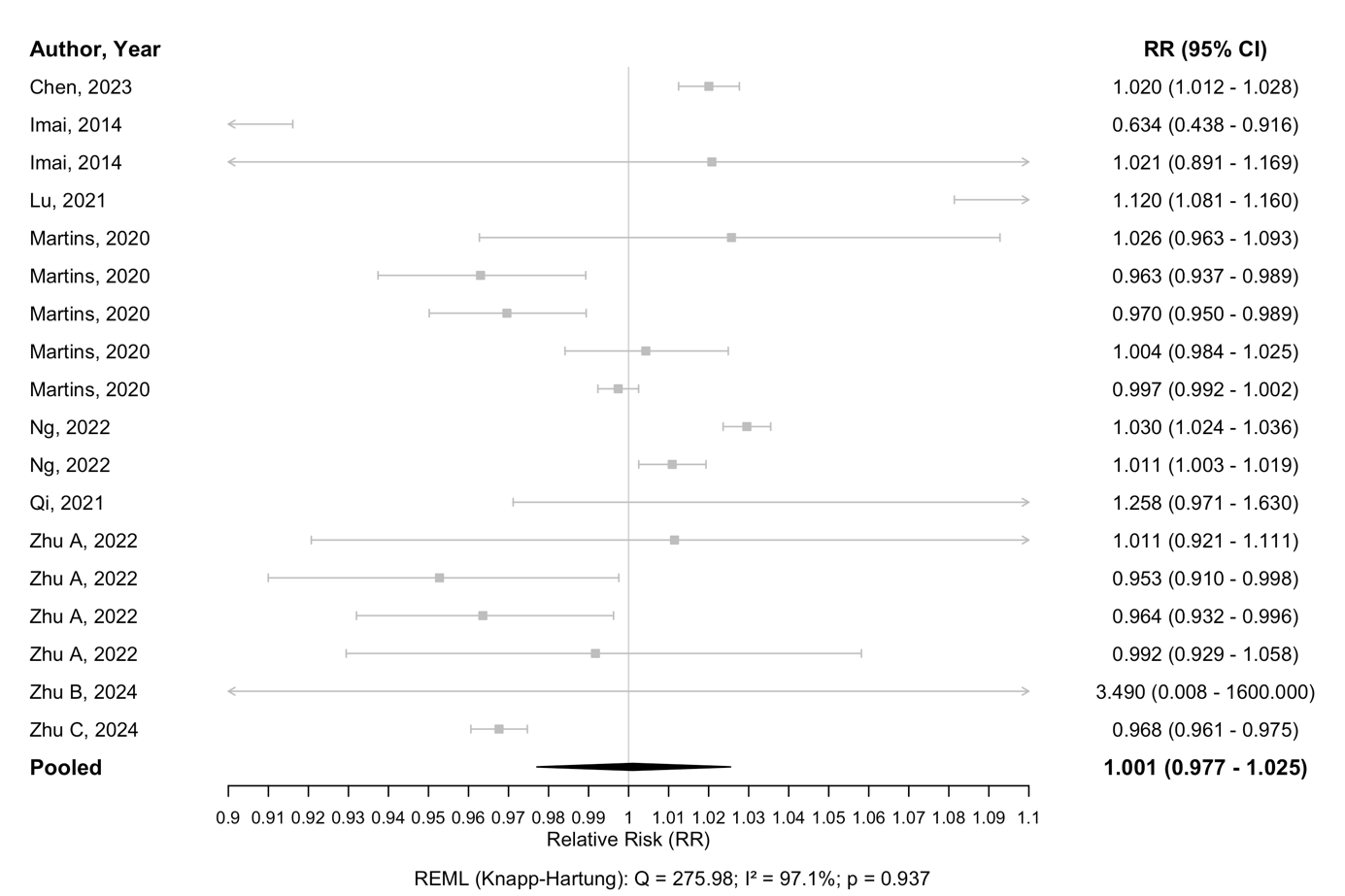


**Figure S10**: Results were presented as relative risks (RR) and 95% confidence intervals (CIs). RE: Random Effects. Q: Cochran’s Q-Statistics. pr_low: lower bound of the 95% prediction interval. pr_up: upper bound of the 95% prediction interval.

**Supplementary Figure S11**. Findings from sensitivity analysis for a random-effects meta-analysis excluding Zhu C, showing RR and 95% CIs for influenza incidence, corresponding to a change per 1 hour increase in sunshine duration.


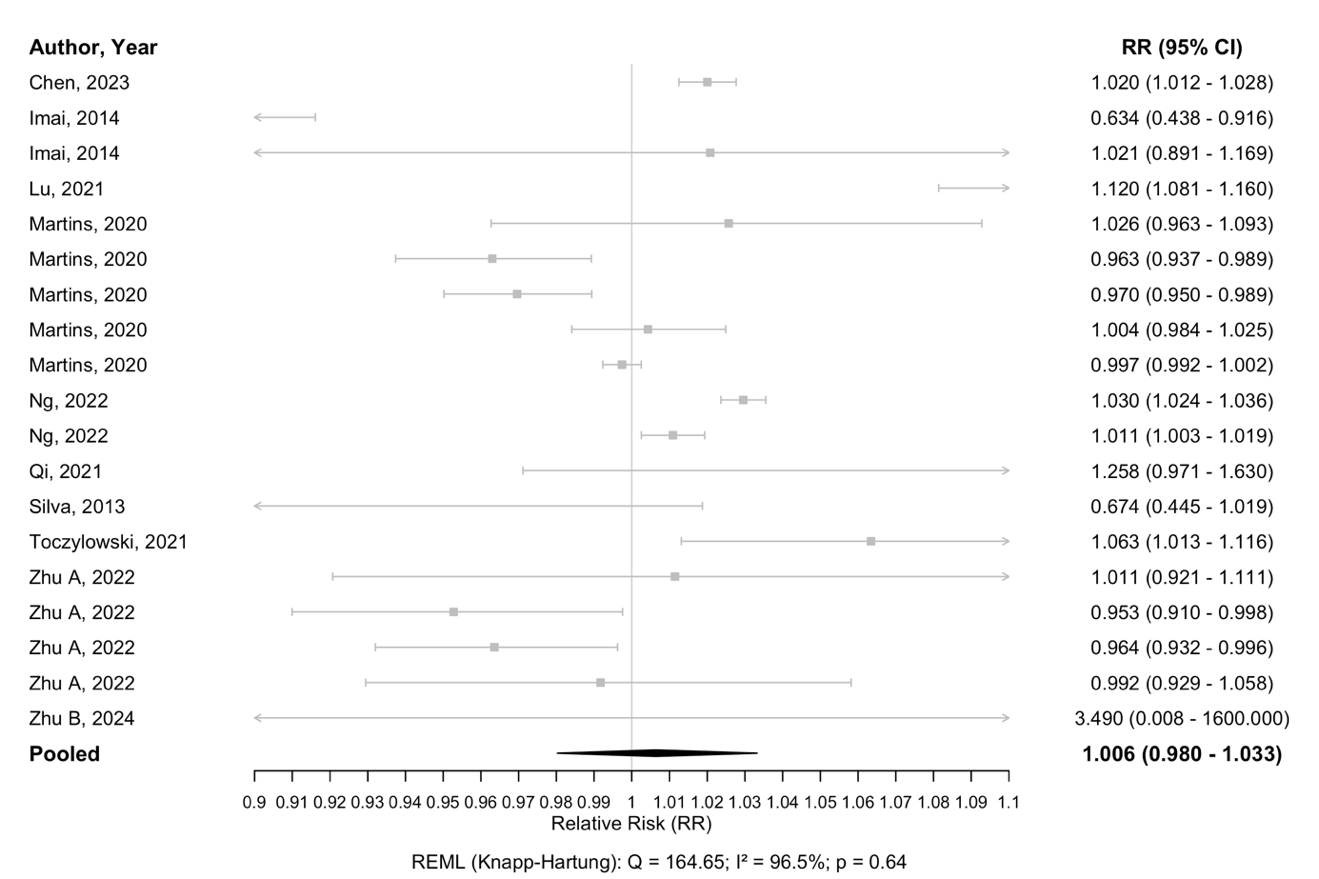


**Figure S11**: Results were presented as relative risks (RR) and 95% confidence intervals (CIs). RE: Random Effects. Q: Cochran’s Q-Statistics. pr_low: lower bound of the 95% prediction interval. pr_up: upper bound of the 95% prediction interval.

**Supplementary Figure S12**. Funnel plot for sunshine duration of study precision (standard error) against effect size (log relative risk). Each point represents an individual study included in the meta-analysis.


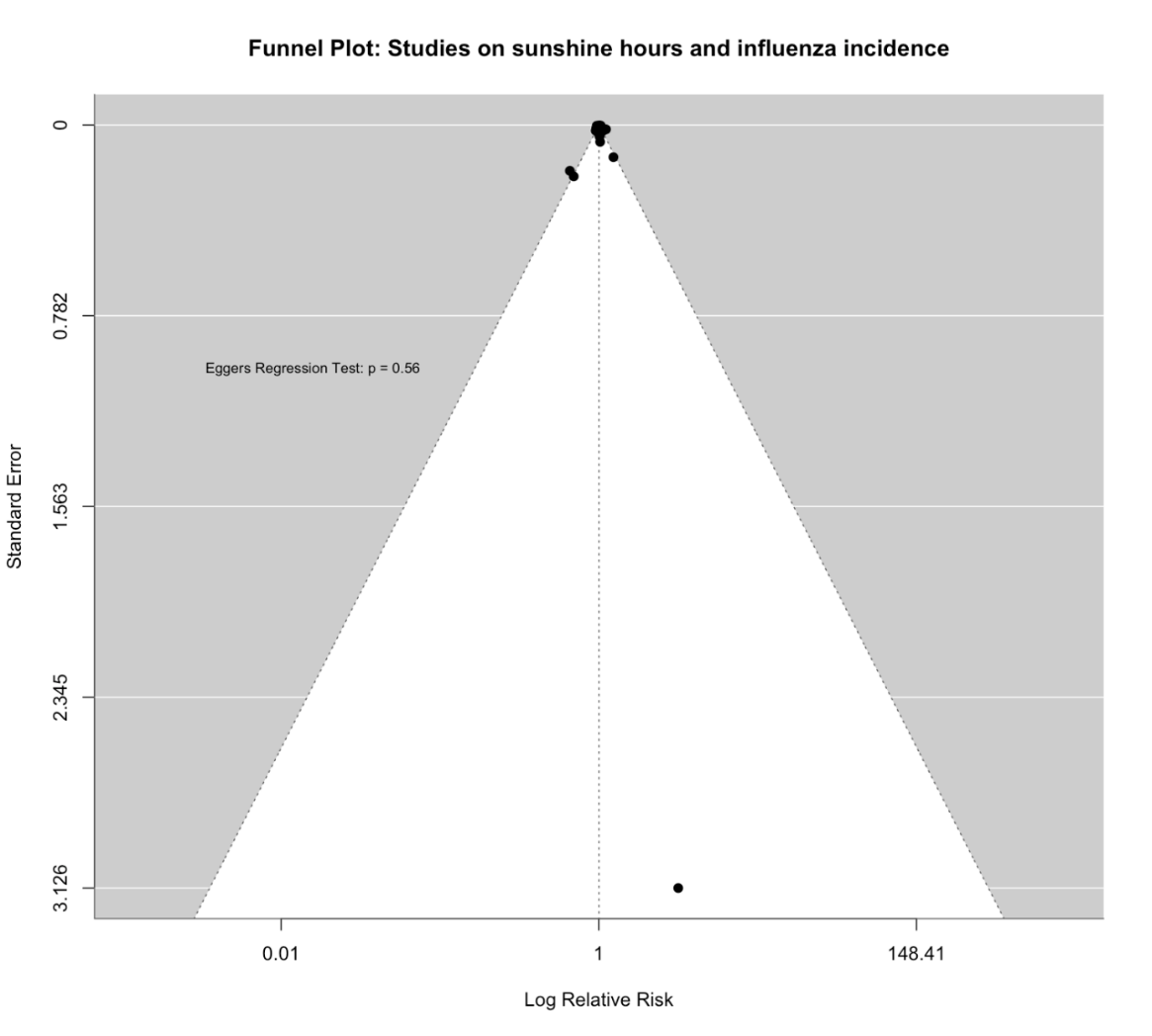


**Supplementary Figure S13.** Findings from univariate meta-regressions for a random-effects meta-analysis showing RR and 95% CIs for influenza incidence, corresponding to a change per 1 hour increase in sunshine duration.


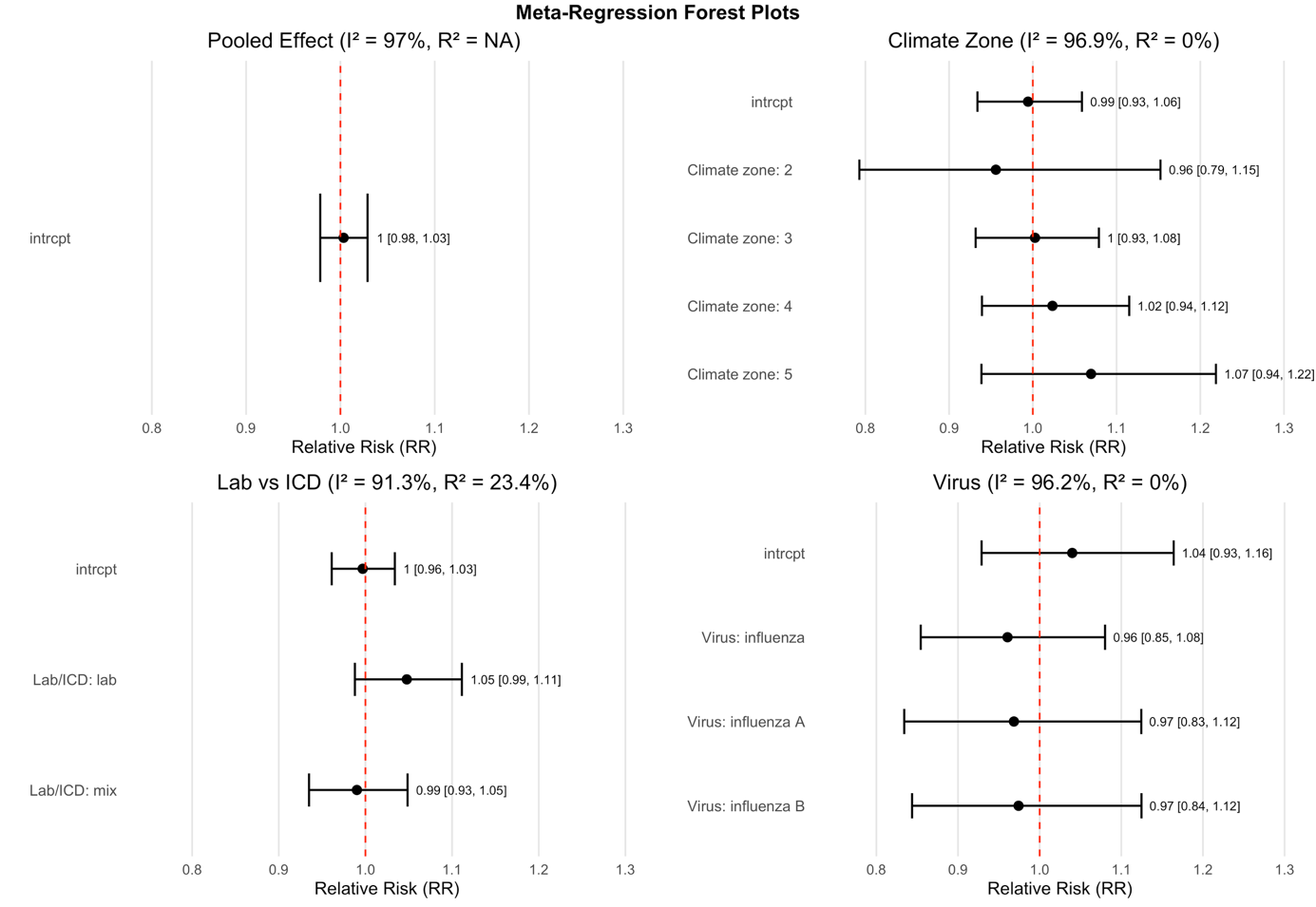


**Figure S13**. Results were presented as RR and 95% confidence intervals (CIs). Intercept for climate zone, Lab vs ICD, and Virus are as follows Climate zone: 1, ICD, and Virus: ILI.

**Supplementary Table S4**. Köppen-Geiger Classifications used for sunshine hours meta-regression.

| **Climate Region (Köppen-Geiger Classificiation)** | **Description** | **Locations** |
| --- | --- | --- |
| **1. Tropical humid (Af / Am)** | Equatorial rainforest or tropical monsoon climate hot and humid year-round with high rainfall. | Amazonas State (Brazil), Northern Maranhão State (Brazil), Coastal Ceará State (Brazil) |
| **2. Tropical monsoonal / savanna transition (Am / Aw)** | Distinct wet and dry seasons; still hot year-round. | Kamalapur (Bangladesh) |
| **3. Subtropical humid to monsoonal (Cwa / Cfa / Aw)** | Warm to hot summers, mild winters; influenced by monsoon rains or subtropical humidity. | Nanping (China), Xiamen (China), Fuzhou (China), Longyan (China), Guangzhou (China), Chongqing (China), Macau (China) |
| **4. Temperate humid (Cfa / Cfb)** | Warm to hot summers, mild to cool winters; evenly distributed precipitation. | Brisbane (Australia), Porto Alegre (Brazil), Southern Paraná (Brazil), Espírito Santo (Brazil) |
| **5. Cool temperate / humid continental (Dfb)** | Warm summers, cold winters, moderate to high precipitation year-round. | Białystok (Poland) |

| **Supplementary Materials S5 – Prisma Checklist** | | |  |
| --- | --- | --- | --- |
| Section and Topic | Item # | Checklist item | Location where item is reported |
| TITLE | | |  |
| Title | 1 | Identify the report as a systematic review. | 1 |
| ABSTRACT | | |  |
| Abstract | 2 | See the PRISMA 2020 for Abstracts checklist. | 2 |
| INTRODUCTION | | |  |
| Rationale | 3 | Describe the rationale for the review in the context of existing knowledge. | 3 |
| Objectives | 4 | Provide an explicit statement of the objective(s) or question(s) the review addresses. | 3 |
| METHODS | | |  |
| Eligibility criteria | 5 | Specify the inclusion and exclusion criteria for the review and how studies were grouped for the syntheses. | 4 |
| Information sources | 6 | Specify all databases, registers, websites, organizations, reference lists and other sources searched or consulted to identify studies. Specify the date when each source was last searched or consulted. | 4 |
| Search strategy | 7 | Present the full search strategies for all databases, registers and websites, including any filters and limits used. | S2 |
| Selection process | 8 | Specify the methods used to decide whether a study met the inclusion criteria of the review, including how many reviewers screened each record and each report retrieved, whether they worked independently, and if applicable, details of automation tools used in the process. | 4 |
| Data collection process | 9 | Specify the methods used to collect data from reports, including how many reviewers collected data from each report, whether they worked independently, any processes for obtaining or confirming data from study investigators, and if applicable, details of automation tools used in the process. | 4 |
| Data items | 10a | List and define all outcomes for which data were sought. Specify whether all results that were compatible with each outcome domain in each study were sought (e.g. for all measures, time points, analyses), and if not, the methods used to decide which results to collect. | 4-5 |
|  | 10b | List and define all other variables for which data were sought (e.g. participant and intervention characteristics, funding sources). Describe any assumptions made about any missing or unclear information. | 4-5 |
| Study risk of bias assessment | 11 | Specify the methods used to assess risk of bias in the included studies, including details of the tool(s) used, how many reviewers assessed each study and whether they worked independently, and if applicable, details of automation tools used in the process. | 5 |
| Effect measures | 12 | Specify for each outcome the effect measure(s) (e.g. risk ratio, mean difference) used in the synthesis or presentation of results. | 5 |
| Synthesis methods | 13a | Describe the processes used to decide which studies were eligible for each synthesis (e.g. tabulating the study intervention characteristics and comparing against the planned groups for each synthesis (item #5)). | 5 |
|  | 13b | Describe any methods required to prepare the data for presentation or synthesis, such as handling of missing summary statistics, or data conversions. | 5 |
|  | 13c | Describe any methods used to tabulate or visually display results of individual studies and syntheses. | 5 |
|  | 13d | Describe any methods used to synthesize results and provide a rationale for the choice(s). If meta-analysis was performed, describe the model(s), method(s) to identify the presence and extent of statistical heterogeneity, and software package(s) used. | 5 |
|  | 13e | Describe any methods used to explore possible causes of heterogeneity among study results (e.g. subgroup analysis, meta-regression). | 5 |
|  | 13f | Describe any sensitivity analyses conducted to assess robustness of the synthesized results. | 5 |
| Reporting bias assessment | 14 | Describe any methods used to assess risk of bias due to missing results in a synthesis (arising from reporting biases). | n/a |
| Certainty assessment | 15 | Describe any methods used to assess certainty (or confidence) in the body of evidence for an outcome. | 5 |
| RESULTS | | |  |
| Study selection | 16a | Describe the results of the search and selection process, from the number of records identified in the search to the number of studies included in the review, ideally using a flow diagram. | 5-6 |
|  | 16b | Cite studies that might appear to meet the inclusion criteria, but which were excluded, and explain why they were excluded. | n/a |
| Study characteristics | 17 | Cite each included study and present its characteristics. | 8-11 |
| Risk of bias in studies | 18 | Present assessments of risk of bias for each included study. | 8-11 |
| Results of individual studies | 19 | For all outcomes, present, for each study: (a) summary statistics for each group (where appropriate) and (b) an effect estimate and its precision (e.g. confidence/credible interval), ideally using structured tables or plots. | 6 |
| Results of syntheses | 20a | For each synthesis, briefly summarise the characteristics and risk of bias among contributing studies. | 12 |
|  | 20b | Present results of all statistical syntheses conducted. If meta-analysis was done, present for each the summary estimate and its precision (e.g. confidence/credible interval) and measures of statistical heterogeneity. If comparing groups, describe the direction of the effect. | 14-15 |
|  | 20c | Present results of all investigations of possible causes of heterogeneity among study results. | 14-16 |
|  | 20d | Present results of all sensitivity analyses conducted to assess the robustness of the synthesized results. | 8, S1 |
| Reporting biases | 21 | Present assessments of risk of bias due to missing results (arising from reporting biases) for each synthesis assessed. | S12 |
| Certainty of evidence | 22 | Present assessments of certainty (or confidence) in the body of evidence for each outcome assessed. | 15-16 |
| DISCUSSION | | |  |
| Discussion | 23a | Provide a general interpretation of the results in the context of other evidence. | 17-18 |
|  | 23b | Discuss any limitations of the evidence included in the review. | 18 |
|  | 23c | Discuss any limitations of the review processes used. | 18 |
|  | 23d | Discuss implications of the results for practice, policy, and future research. | 18-19 |
| OTHER INFORMATION | | |  |
| Registration and protocol | 24a | Provide registration information for the review, including register name and registration number, or state that the review was not registered. | 5 |
|  | 24b | Indicate where the review protocol can be accessed, or state that a protocol was not prepared. | 5 |
|  | 24c | Describe and explain any amendments to information provided at registration or in the protocol. | 5 |
| Support | 25 | Describe sources of financial or non-financial support for the review, and the role of the funders or sponsors in the review. | 19 |
| Competing interests | 26 | Declare any competing interests of review authors. | 19 |
| Availability of data, code and other materials | 27 | Report which of the following are publicly available and where they can be found: template data collection forms; data extracted from included studies; data used for all analyses; analytic code; any other materials used in the review. | S2-6 |

*From:*  Page MJ, McKenzie JE, Bossuyt PM, Boutron I, Hoffmann TC, Mulrow CD, et al. The PRISMA 2020 statement: an updated guideline for reporting systematic reviews. BMJ 2021;372:n71. doi: 10.1136/bmj.n71
